# Biomineralized Materials as Model Systems for Structural Composites: Intracrystalline Structural Features and Their Strengthening and Toughening Mechanisms

**DOI:** 10.1002/advs.202103524

**Published:** 2022-03-22

**Authors:** Zhifei Deng, Zian Jia, Ling Li

**Affiliations:** ^1^ Department of Mechanical Engineering Virginia Polytechnic Institute of Technology and State University Blacksburg VA 24060 USA

**Keywords:** biominerals, calcium carbonate, hydroxyapatite, intracrystalline structure, mechanical properties

## Abstract

Biomineralized composites, which are usually composed of microscopic mineral building blocks organized in 3D intercrystalline organic matrices, have evolved unique structural designs to fulfill mechanical and other biological functionalities. While it has been well recognized that the intricate architectural designs of biomineralized composites contribute to their remarkable mechanical performance, the structural features within and corresponding mechanical properties of individual mineral building blocks are often less appreciated in the context of bio‐inspired structural composites. The mineral building blocks in biomineralized composites exhibit a variety of salient intracrystalline structural features, such as, organic inclusions, inorganic impurities (or trace elements), crystalline features (e.g., amorphous phases, single crystals, splitting crystals, polycrystals, and nanograins), residual stress/strain, and twinning, which significantly modify the mechanical properties of biogenic minerals. In this review, recent progress in elucidating the intracrystalline structural features of three most common biomineral systems (calcite, aragonite, and hydroxyapatite) and their corresponding mechanical significance are discussed. Future research directions and corresponding challenges are proposed and discussed, such as the advanced structural characterizations and formation mechanisms of intracrystalline structures in biominerals, amorphous biominerals, and bio‐inspired synthesis.

## Graphical abstract

The mineral building blocks in biomineralized composites exhibit salient intracrystalline structural features, such as organic inclusions, trace elements, varying crystalline features, residual stress/strain, and twinning, which significantly modifies the mechanical properties of biogenic minerals. This review discusses recent progress in elucidating the intracrystalline structural features of three common biomineral systems (calcite, aragonite, and hydroxyapatite) and their corresponding mechanical significance.

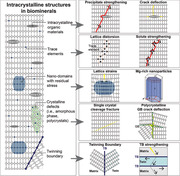

## 1 Introduction

In nature, organisms often rely on stiff and strong skeletal elements for structural support and other functional requirements for survival.^[^ ^1^ ^]^ Typical examples include endoskeletons (internal to body) for body support and protection of internal organs and exoskeletons (external to body) for protection from the environment and predators’ attacks.^[^ ^2^ ^]^ In order to achieve desired mechanical properties, such as stiffness, strength, and fracture resistance, many of these biological skeletal elements are mineralized. The complex hierarchical organizations of biomineral building blocks with organic components allow organisms to build robust composites for the aforementioned functions. For example, the well‐known “brick‐and‐mortar” structure in nacre, a common composite structure in many mollusk shells, is able to amplify the work of fracture by three orders of magnitude compared to its mineral phase (aragonite).^[^ ^3^ ^]^ The composite design strategies and associated strengthening and toughening mechanisms have been extensively studied, with the aim of developing bio‐inspired high‐performance composite materials.^[^ ^2^, ^4^, ^5^ ^]^

While the structural design principles at composite level are well recognized in the literature, the intracrystalline structure and mechanical properties of individual biomineral building blocks are often less considered in the investigations of biomineral composites.^[^ ^2^, ^4^, ^5^ ^]^ Although the minerals formed by organisms, that is, biominerals or biogenic minerals, have similar compositions to the geological counterparts, they exhibit some important distinctions.^[^ ^1^ ^]^ For example, biogenic minerals are themselves composite materials, as they often integrate a small amount of organic materials within the mineral matrix, despite that individual biomineral building blocks often diffract as single crystals.^[^ ^6^, ^7^ ^]^ Biogenic minerals also exhibit lattice distortions in comparison to their synthetic or geological counterparts.

If we consider biomineral composites as analogs to engineering structural materials/composites, the individual building blocks in biomineral composites correspond to the grains/reinforcing phases in engineering materials. The intracrystalline features within individual biomineral building blocks, such as, impurities, residual stress/strain, crystallinity, and grain morphologies, have profound effects in controlling the mechanical properties of biominerals. In this review, we provide a systematic overview of the current knowledge and recent advances in this area. Specifically, we focus on the intracrystalline structures within individual biomineral building blocks and their associated toughening/strengthening mechanisms. The analogy and difference between biominerals and engineering structural materials are reviewed. In addition, we discuss the open questions and challenges in further understanding the mechanical designs of biogenic minerals. We hope this review can provide collective insights for developing novel bio‐inspired materials through engineering the intracrystalline structural features at individual building block level.

## 2 Biogenic Minerals: General Characteristics

### 2.1 Biomineral Composites versus Biomineral Building Blocks

Currently, over 60 biogenic minerals have been identified in different organisms. Typical examples include carbonates and silica (widely found in protists, plants, and animals), phosphates (found in monerans and animals), and oxalates (found in fungi, plants, and animals).^[^ ^1^ ^]^ Calcite and aragonite, being two polymorphs of calcium carbonates, are the most essential minerals in constructing skeletal elements in various invertebrate groups, such as echinoderms, mollusks, and arthropods;^[^ ^1^, ^8^ ^]^ while hydroxyapatite (HAP) crystals are commonly found as the building units in bone and teeth of vertebrates.^[^ ^2^, ^9^, ^10^ ^]^ Therefore, in this review, we primarily focus on these three most common biogenic minerals.

**Figure 1:**
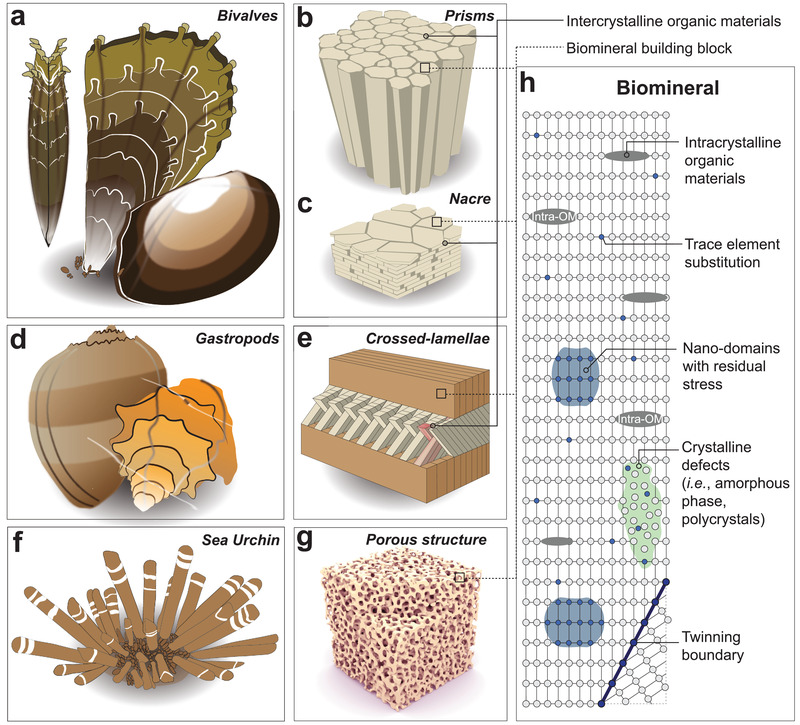
Overview of biomineral skeletal elements and intracrystalline structural features within the individual building blocks. a‐c) Schematic illustrations of a) bivalve shells as examples of densely packed biomineral composites, and their typical microstructures of b) prism‐based and c) platelet‐based composites (nacre), where the mineral building blocks are separated by intercrystalline organics. d,e) Schematic illustrations of d) gastropod shells as examples of another type of densely packed biomineral composites, and e) the typical crossed‐lamellae microstructure with fiber‐based building blocks. f,g) Schematic illustrations of f) sea urchin spines as examples of porous biomineral skeletal elements, and their microstructure g) open cell bicontinuous biomineral foams. h) Schematic illustration of the intracrystalline structural features of the mineral building blocks (lattice), including intracrystalline organic materials (intra‐OMs), trace element substitution, nano‐heterogeneity domains, crystalline defects, and twinning boundaries.

Based on the composite‐level structural features, biomineral composites can be broadly classified into two groups: i) Solid composites composed of densely packed mineral building blocks (e.g., mollusk shells in **Figure** **1**a,d), and ii) porous biomineralized skeletal elements with a bicontinuous structure (e.g., porous skeletons of sea urchins in Figure 1f). The first type of biomineral composites is well‐known for their hierarchical designs across multiple scales, where the microscopic biomineral units are joined together by thin organic interfaces. The mineral units are defined as the basic building blocks, such as the prisms in the prismatic layer (Figure 1b), tablets in the nacre composites (Figure 1c), and aragonite fibers/lamellae (3rd‐order lamellae)^[^ ^11^ ^]^ in the crossed‐lamellar structure (Figure 1e) in mollusk shells. The organic layers between the building blocks are referred as the intercrystalline organic materials (inter‐OMs), as shown in Figure 1b,c,e. In contrast, the second type of biomineral composites does not have interconnected inter‐OMs (Figure 1g); in sea urchins, for example, each spine diffracts as a single crystal under X‐ray diffraction (XRD) and thus can be considered as a single large building block.^[^ ^6^, ^7^ ^]^

There have been many studies on the composite‐level structures, properties, and deformation mechanisms of biological composites, and representative reviews can be found in refs. [2, 4, 12, 13]. A general consensus of this body of work is that the structural hierarchies via intricate organization of biomineral building blocks and inter‐OMs contribute to the enhanced toughness significantly.^[^ ^3^, ^11^ ^]^ Furthermore, extensive efforts have been made on translating the hierarchical structural designs to bio‐inspired composites.^[^ ^14^, ^15^, ^16^, ^17^, ^18^ ^]^ A further discussion on recent advances in the understanding of the 3D structural organizations and corresponding composite‐level design strategies in biomineral composites can be found in our companion perspective paper.^[^ ^19^ ^]^

### 2.2 Mechanical Difference between Biogenic and Geological Minerals

**Figure 2:**
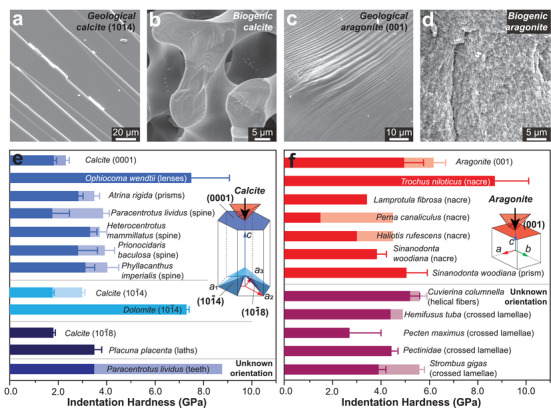
Mechanical difference between biogenic and geological minerals. a,b) Scanning electron microscopy (SEM) images on the fracture surfaces of a) geological calcite and b) biogenic calcite (sea urchin spine, *Heterocentrotus mammillatus*), where the former shows preferred cleavage fracture and the latter shows conchoidal fracture. c,d) Fracture surfaces of c) geological aragonite and d) biogenic aragonite (*Sinanodonta woodiana*). e,f) Nanoindentation hardness of biogenic and geological e) calcite^[^ ^23^, ^24^, ^25^, ^26^, ^27^, ^28^ ^]^ and f) aragonite^[^ ^29^, ^30^, ^31^, ^32^, ^33^, ^34^, ^35^ ^]^ along different crystallographic orientations, where the overlapping bars shaded with lighter colors indicate different results for the same species. Corresponding crystallographic planes are shown in the inset illustrations. a,c) Reproduced with permission.^[^ ^35^ ^]^ Copyright 2021, American Chemical Society.

It has been long recognized that biogenic minerals have different fracture characteristics from geological pure forms. The geological minerals often fracture along the cleavage planes (**Figure** **2**a), while the biogenic minerals exhibit conchoidal fracture resembling amorphous glass (Figure 2b). The earliest observation of conchoidal fracture in biominerals was found in calcareous sponge, which dated back to 1869 by Carter,^[^ ^20^ ^]^ and similar fracture characteristics were observed in echinoderm calcite.^[^ ^6^, ^7^, ^21^ ^]^ The biogenic and geological aragonite also fracture differently (Figure 2c,d).

Biogenic and geological minerals also exhibit different mechanical properties, such as stiffness and hardness. For example, the bending stiffness of the calcitic spicules of echinoid larvae is 2–4 times lower than that of geological calcite.^[^ ^22^ ^]^ Nanoindentation reveals that biogenic calcite has a higher hardness than geological calcite (measured on different crystallographic planes, Figure 2e).^[^ ^23^, ^24^, ^25^, ^26^, ^27^, ^28^ ^]^ In contrast, biogenic aragonite exhibits (mostly) lower hardness compared to geological aragonite (Figure 2f).^[^ ^29^, ^30^, ^31^, ^32^, ^33^, ^34^, ^35^ ^]^ Such difference might correlate with the grain sizes and the crystalline features. Biogenic calcite usually exists as micro‐sized single crystals, such as the calcite prisms (20–50 µm) in mollusk shells and porous skeletons in echinoderms (branch thickness of 15–35 µm), so that the nanoindentation (usually with indentation depths of hundreds of nanometers) is not influenced by boundary effects. In comparison, biogenic aragonite often exists as smaller building blocks via aggregation of nanograins (e.g., nacre tablets, thickness of 1–2 µm, composed of 3‐10 nm nanograins),^[^ ^30^ ^]^ which impose strong boundary effects, and the extensive intracrystalline organics may further degrade the hardness.

### 2.3 Intracrystalline Structural Features of Biogenic Minerals

The mechanical difference between biogenic and geological minerals is extremely intriguing, considering that many biomineral building blocks diffract as single crystals under electron and X‐ray beams.^[^ ^6^, ^7^ ^]^ The different mechanical properties suggest that the biogenic minerals should possess different internal nanoscopic structures in comparison to their geological or pure forms. Indeed, many recent studies have revealed the subtle structural modifications and defects within individual biomineral building blocks. These structural defects include intracrystalline organic materials (intra‐OMs), trace element substitution, crystalline defects (amorphous phase, polycrystalline, splitting crystal, nanograins, etc.), inhomogeneous nano‐domains, and twinning (Figure 1h). Here, the intra‐OMs refer to those organic materials enclosed inside the biomineral building blocks (at intracrystalline level), in contrast to the inter‐OMs defined as the organic interfaces between adjacent building blocks (at composite level).

**Figure 3:**
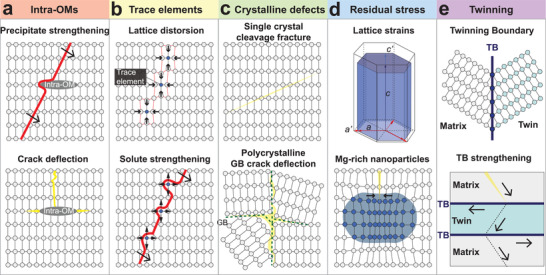
Overview of the strengthening and toughening mechanisms of the intracrystalline structural features within biominerals. a) Intracrystalline organic materials (intra‐OM, gray ellipse) inhibit dislocation motion (red line, direction indicated by arrows) and promote deflection of cracks (yellow lines). b) Trace element substitutions (solid blue circles) introduce local lattice distortion and solute strengthening to prevent dislocation motion. c) Comparison of cleavage fracture in single crystal and crack deflection at grain‐boundaries (GB) in polycrystalline minerals. d) Residual strain and stress induced by crystal lattice distortion and Mg‐rich nanodomains^[^ ^92^ ^]^ in biominerals. e) Twinning boundary (TB) strengthening where mobile and/or sessile dislocations could be generated either in neighboring domains or at TBs, and cracks are deflected and impeded at the TBs.^[^ ^224^ ^]^

It is well‐known that the structural defects within individual grains in engineering metals and ceramics have significant effects in controlling the mechanical properties.^[^ ^36^ ^]^ In analogue to individual grains in engineering materials, the intracrystalline structural features in biomineral building blocks are expected to have similar mechanical contributions. For example, the incorporation of intra‐OMs has been shown to strengthen the biominerals by dislocation impediment (**Figure** **3**a), similar to the effect of nanoscale secondary‐phase precipitation in engineering materials. Additional toughening is also induced by crack deflection along the intra‐OMs. The trace element substitutions in crystal lattice induce local lattice distortions (Figure 3b) and residual stress/strain (Figure 3d), leading to strengthening effect. The crystalline features also control the cracking behaviors. For example, cracks tend to propagate along the grain boundaries (GB) in polycrystalline minerals, while cleavage cracks are often found in single‐crystal minerals (Figure 3c). In addition, twinning boundaries represent another factor that provides crack impeding and deflection in the biomineral building blocks (Figure 3e). In this review article, we summarize the recent advances in understanding these intracrystalline structural features of biominerals and discuss their strengthening and toughening mechanisms.

In the following sections, we focus on five aspects of intracrystalline structural features found in biogenic minerals, including intra‐OMs (Section 3), substitution of trace elements in the crystal lattice (Section 4), crystalline features and defects (Section 5), residual strain/stress (Section 6), and twinning (Section 7). Each type of intracrystalline features has distinctive contributions to the mechanical properties of biominerals, as summarized in Figure 3; they also work in synergy to promote combined high toughness and strength. In each section, we present the recent advances in characterizing these structural features and show how the intracrystalline defects affect the properties of biominerals. **Table** **1** summarizes the calcium carbonate‐based biomineral building blocks discussed in this review. Note that there are different types of biomineral composites and building blocks in the hard skeletons of one organism; for instance, many bivalve shells consist of an outer prismatic layer (prisms) and an inner nacreous layer (tablets).

**Table 1:** Calcium carbonate‐based biomineral building blocks

Animal/Class	Species	Building blocks (Crystal polymorphs)	Relevant topics and references
Bivalve shell	*Acanthocardia tuberculata*	Crossed‐lamellar (aragonite)	Residual strain.^[^ ^71^, ^72^, ^73^ ^]^
	*Arctica islandica*	Prisms (aragonite)	Trace elements.^[^ ^106^ ^]^
	*Atrina rigida*	Prisms (calcite)	Intra‐OMs;^[^ ^28^, ^40^, ^44^, ^55^, ^58^ ^]^ Trace elements;^[^ ^68^, ^99^ ^]^ Residual strain.^[^ ^68^, ^99^, ^186^ ^]^
	*Atrina serrata*	Prisms (calcite)	Intra‐OMs.^[^ ^43^ ^]^
	*Atrina pectinata*	Prisms (calcite)	Intra‐OMs.^[^ ^50^ ^]^
	*Crassostrea gigas*	Prisms (calcite)	Trace elements;^[^ ^68^ ^]^ Residual strain.^[^ ^68^, ^186^ ^]^
		Larval shell (aragonite + ACC)	Crystalline features (ACC).^[^ ^169^ ^]^
	*Mercenaria*	Larval shell (aragonite + ACC)	Crystalline features (ACC).^[^ ^169^ ^]^
	*Meretrix lamarckii*	Crossed‐lamellar (aragonite)	Twinning.^[^ ^239^ ^]^
	*Ostrea edulis*	Prisms (calcite)	Trace elements;^[^ ^68^ ^]^ Residual strain.^[^ ^68^, ^186^ ^]^
	*Perna canaliculus*	Nacre (aragonite)	Intra‐OMs;^[^ ^46^ ^]^ Residual strain.^[^ ^72^, ^73^, ^213^, ^214^ ^]^
	*Pinctada fucata*	Prisms (calcite)	Intra‐OMs;^[^ ^50^, ^66^ ^]^ Crystalline features (splitting crystal).^[^ ^134^, ^277^ ^]^
	*Pinctada maxima*	Nacre (aragonite)	Intra‐OMs;^[^ ^47^, ^78^ ^]^ Crystalline features (nanograins).^[^ ^201^ ^]^
	*Pinctada margaritifera*	Prisms (calcite)	Trace elements;^[^ ^105^ ^]^ Crystalline features (splitting crystal).^[^ ^135^, ^187^, ^191^, ^193^ ^]^
		Nacre (aragonite)	Trace elements.^[^ ^105^ ^]^
	*Pinctada nigra*	Prisms (calcite)	Crystalline features (splitting crystal).^[^ ^192^ ^]^
	*Pinna nobilis*	Prisms (calcite)	Intra‐OMs;^[^ ^52^, ^53^, ^61^ ^]^ Trace elements;^[^ ^68^ ^]^ Residual strain;^[^ ^68^, ^186^ ^]^ Crystalline features (nanograins).^[^ ^142^, ^189^ ^]^
		Nacre (aragonite)	Crystalline features (nanograins);^[^ ^199^ ^]^ Twinning.^[^ ^139^ ^]^
	*Placuna placenta*	Foliated laths (calcite)	Twinning.^[^ ^206^ ^]^
	*Pteria hirundo*	Nacre (aragonite)	Twinning.^[^ ^139^ ^]^
	*Saxidomus purpuratus*	Crossed‐lamellar (aragonite)	Twinning.^[^ ^240^ ^]^
	*Sinanodonta woodiana*	Prisms (aragonite)	Crystalline features (polycrystal).^[^ ^35^ ^]^
	*Unio pictorum*	Nacre (aragonite)	Intra‐OMs.^[^ ^278^ ^]^
Bivalve hinge	*Mya arenaria*	Straight fibers (aragonite)	Twinning.^[^ ^244^ ^]^
	*Neotrigonia sp*.	Straight fibers (aragonite)	Twinning.^[^ ^246^ ^]^
	*Pinctada fucata*	Straight fibers (aragonite)	Twinning.^[^ ^245^ ^]^
	*Spisula solidissima*	Straight fibers (aragonite)	Twinning.^[^ ^244^ ^]^
Gastropod shell	*Clio pyramidata*	Helical fibers (aragonite)	Intra‐OMs; Twinning.^[^ ^48^ ^]^
	*Cuvierina columnella*	Helical fibers (aragonite)	Twinning.^[^ ^237^, ^238^ ^]^
	*Haliotis discus hannai*	Nacre (aragonite)	Twinning.^[^ ^234^ ^]^
	*Haliotis laevigata*	Nacre (aragonite)	Intra‐OMs;^[^ ^65^ ^]^ Crystalline features (nanograins^[^ ^139^ ^]^ and ACC^[^ ^170^ ^]^).
	*Haliotis rufescens*	Prisms (calcite)	Trace elements;^[^ ^68^ ^]^ Residual strain.^[^ ^68^, ^186^ ^]^
		Nacre (aragonite)	Crystalline features (nanograins^[^ ^30^, ^85^, ^138^, ^200^, ^201^ ^]^ and ACC^[^ ^171^ ^]^); Twinning.^[^ ^247^ ^]^
	*Murex troscheli*	Crossed‐lamellar (aragonite)	Twinning.^[^ ^241^ ^]^
	*Strombus decorus persicus*	Crossed‐lamellar (aragonite)	Residual stress.^[^ ^72^ ^]^
	*Phorcus turbinatus*	Nacre (aragonite)	Crystalline features (nanograins).^[^ ^140^ ^]^
	*Strombus gigas*	Crossed‐lamellar (aragonite)	Twinning.^[^ ^243^ ^]^
Monoplacophoran shell	*Micropilina arntzi*	Foliated laths (aragonite)	Twinning.^[^ ^236^ ^]^
Sea urchin	*Authoeidaris Erassispina*	Spines: Porous (calcite)	Crystalline features (nanograins + ACC).^[^ ^141^ ^]^
	*Echinus esculentus*	Spines: Porous (calcite)	Trace elements.^[^ ^95^ ^]^
	*Heterocentrotus mammillatus*	Spines: Porous (calcite)	Trace elements.^[^ ^95^ ^]^
	*Lytechinus variegatus*	Spines: Porous (calcite)	Trace elements.^[^ ^95^ ^]^
	*Paracentrotus lividus*	Spines: Porous (calcite)	Intra‐OMs;^[^ ^41^, ^42^, ^43^, ^51^, ^58^, ^59^, ^64^ ^]^ Trace elements;^[^ ^25^, ^95^ ^]^ Crystalline features (ACC).^[^ ^151^, ^185^ ^]^
		Teeth: Composite (calcite)	Trace elements;^[^ ^97^, ^98^ ^]^ Crystalline features (single crystal + polycrystal).^[^ ^98^ ^]^
		Larval spicule (calcite + ACC)	Crystalline features (ACC).^[^ ^167^, ^168^ ^]^
	*Sterechinus antarcticus*	Spines: Porous (calcite)	Trace elements.^[^ ^95^ ^]^
	*Strongylocentrotus franciscanus*	Spines: Porous (calcite)	Trace elements.^[^ ^115^ ^]^
	*Strongylocentrotus purpuratus*	Spines: Porous (calcite)	Trace elements;^[^ ^115^ ^]^ Crystalline features (nanograins and ACC).^[^ ^155^, ^174^ ^]^
		Teeth: Composite (calcite)	Trace elements.^[^ ^103^ ^]^
		Larval spicule (calcite + ACC)	Crystalline features (ACC).^[^ ^152^, ^168^, ^173^ ^]^
Sea star	*Asterias rubens*	Porous (calcite)	Intra‐OMs.^[^ ^63^ ^]^
	*Echinaster spinulosus*	Porous (calcite)	Intra‐OMs.^[^ ^62^ ^]^
	*Pisaster giganteus*	Porous (calcite)	Intra‐OMs;^[^ ^49^ ^]^ Trace elements.^[^ ^95^ ^]^
Brittle star	*Ophiocoma wendtii*	Porous (calcite)	Trace elements;^[^ ^24^ ^]^ Residual strain.^[^ ^24^, ^92^, ^100^ ^]^
Calcareous sponge	*Clathrina contorta*	Triradiate sponge (calcite + ACC)	Crystalline features (ACC).^[^ ^60^, ^161^ ^]^
Coral	*Stylophora pistillata*	Coral skeleton (aragonite)	Crystalline features (ACC^[^ ^172^ ^]^ + polycrystal^[^ ^194^, ^195^ ^]^).
Coccolithophores	*Emiliania huxleyi*	Coccolith (calcite)	Intra‐OMs; Trace elements.^[^ ^69^ ^]^
	*Pleurochrysis carterae*	Coccolith (calcite)	Intra‐OMs; Trace elements.^[^ ^69^ ^]^

## 3 Intracrystalline Organics (intra‐OMs)

**Figure 4:**
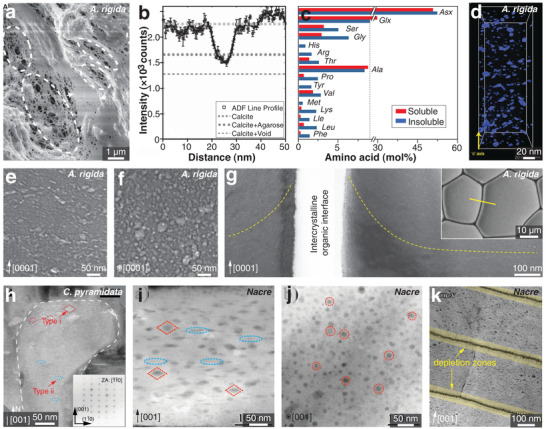
Intracrystalline organic materials: Chemistry, morphologies, and distributions in a–g) biogenic calcite (*A*. *rigida* prisms) and h–j) aragonite (fiber and nacre building blocks). a) SEM image of the intra‐OMs in etched pits of *A*. *rigida* prism. b) ADF‐STEM intensity across an inclusion in *A*. *rigida* prism, in comparison with pure calcite, calcite with void, and calcite with organic materials (agarose). c) Quantitative measurement of different amino acids in the soluble and insoluble intra‐OMs in *A*. *rigida* prism.^[^ ^44^ ^]^ d) 3D visualization of intra‐OMs in *A*. *rigida* based on ADF‐STEM electron tomography. e,f) TEM images on the sections cut e) parallel and f) perpendicular to the length axis of *A*. *rigida* prisms (*c*‐axis [0001] of calcite), where brighter regions denote the intracrystalline inclusions. g) TEM image across the intercrystalline organic interface between *A. rigida* prisms, where the yellow dashed lines indicate the alignment of the inclusions. The yellow line in the inset shows the cut location for the TEM image. h) TEM image of the aragonite fiber building block in *C. pyramidata*, showing two types of intra‐OMs, that is, type i diamond‐shaped inclusions (red dashed rhombus) and type ii elongated inclusions (blue dashed ellipse). i,j) ADF‐STEM images of nacre tablets (*Pinctada maxima*) i) parallel and j) perpendicular to the thickness direction (*c*‐axis [001] of aragonite), respectively, where the inclusions show darker contrast compared to the aragonite matrix. k) Random distribution of intra‐OMs and depletion zones (yellow‐shaded regions with no inclusions) in nacre tablets (*Perna canaliculus*). a,b,d) Reproduced with permission.^[^ ^40^ ^]^ Copyright 2011, Wiley‐VCH. e–g) Reproduced with permission.^[^ ^28^ ^]^ Copyright 2020, Springer Nature. h) Reproduced with permission.^[^ ^48^ ^]^ Copyright 2015, Springer Nature. i,j) Reproduced with permission.^[^ ^47^ ^]^ Copyright 2015, Royal Society of Chemistry. k) Reproduced with permission.^[^ ^46^ ^]^ Copyright 2012, American Chemical Society.

Intra‐OMs refer to the organic materials distributed within individual biomineral building blocks, in contrast to inter‐OMs located at the interfaces between adjacent biomineral units.^[^ ^37^ ^]^ Early observations of the inter‐ and intra‐OMs was achieved by decalcification experiments in bivalve nacre.^[^ ^38^ ^]^ Later in 1970s, transmission electron microscopy (TEM) was utilized to examine the intra‐OMs in nacre tablets, which revealed their bubbly and frothy morphologies.^[^ ^39^ ^]^ Recent advances in TEM‐based techniques have contributed to further understanding of intra‐OM compositions. For example, the annular dark‐field scanning transmission electron microscopy (ADF‐STEM) demonstrated that the inclusions (dashed ellipses in **Figure** **4**a) in the *Atrina rigida* prisms contain organic materials rather than voids (Figure 4b).^[^ ^40^ ^]^ Berman et al. further suggested that intra‐OMs are selectively occluded into specific crystallographic planes of the mineral matrix,^[^ ^41^ ^]^ which might be responsible for biominerals’ unique fracture characteristics^[^ ^42^ ^]^ and crystal textures.^[^ ^43^ ^]^ It is now generally accepted that the intra‐OMs are widely distributed in different types of biomineral building blocks, including prisms (calcite),^[^ ^40^, ^44^ ^]^ foliated laths (calcite),^[^ ^45^ ^]^ nacre tablets (aragonite),^[^ ^46^, ^47^ ^]^ curved nanofibers in pteropod shells (aragonite),^[^ ^48^ ^]^ porous stereom in echinoderm skeletons (calcite),^[^ ^49^ ^]^ etc.

### 3.1 Chemistry of Intracrystalline Organic Materials

The systematic characterization of intra‐OM chemical compositions is of great importance. Previous studies on intra‐OM and inter‐OM chemistry have been focused on the prismatic layers in *A. rigida*,^[^ ^44^ ^]^ *Pinctada fucata* and *Atrina pectinata*,^[^ ^50^ ^]^ and the echinoderm calcite in *Paracentrotus lividus* ^[^ ^51^ ^]^ and *Pisaster giganteus*.^[^ ^49^ ^]^ Typically, biomineral composites exhibit a higher amount of inter‐OMs than intra‐OMs, for example, ≈5 wt% versus 0.4 wt% (weight percentages) in the prismatic layer of *A. rigida*, respectively.^[^ ^44^ ^]^ The intra‐OMs consist mostly of proteins and chitin,^[^ ^44^ ^]^ while the specific concentrations vary in different species. Figure 4c shows detailed compositions of amino acids in intra‐OMs from *A. rigida* prisms (in both water‐soluble and insoluble phases),^[^ ^44^ ^]^ which significantly differ from *P. fucata* prisms (27.4% Asx, 10.3% Glx, 17.5% Gly, etc.) and *A. pectinata* prisms (45.6% Asx, 12.8% Glx, 10.8% Gly, etc.).^[^ ^50^ ^]^ It should also be noted that intra‐OMs are reported to bear negative charges,^[^ ^50^, ^52^ ^]^ which attract cations (e.g., Ca^2+^ in calcium‐based minerals) and therefore align themselves to the charged planes in the crystal lattice, for instance, {0001} planes in calcite.^[^ ^53^ ^]^ In addition to the diverse variety of amino acids, chitin morphology is also found to vary in different biomineral systems. For example, while no chitin was found in the inter‐OMs in *A. rigida* prisms,^[^ ^44^ ^]^ chitin sheets were found in the inter‐OM scaffold between nacre lamellae.^[^ ^54^ ^]^ Moreover, the study by Li et al. revealed that chitin resided in calcitic prisms (*A. rigida*) as a fibrous network coated with proteinaceous globular structures.^[^ ^44^ ^]^

While the intra‐OM compositions vary significantly in different biogenic calcite, the nanoindentation properties are rather consistent (indentation results on {0001} in Figure 2e). This suggests that the compositions of intra‐OMs may have little influence on the mechanical performance. In contrast, it might be the relative percentage and distribution of intra‐OMs that control the mechanical behaviors of biogenic minerals. However, the chemical compositions of intra‐OMs may play a significant role in controlling the nucleation, growth process, and the formed shape of the biominerals.^[^ ^42^ ^]^ In vitro experiments revealed that the intra‐OMs extracted from different biominerals (sea urchin spines, mollusk shells, calcareous sponge, etc.) could induce distinctive modifications in crystal microstructures and morphologies.^[^ ^50^, ^55^, ^56^, ^57^ ^]^ For example, calcite crystals synthesized in the presence of intra‐OMs extracted from mollusk prisms (*A. rigida*) exhibited flattened morphologies perpendicular to calcite *c*‐axis, while the incorporation of intra‐OMs from sea urchin spines (*P. lividus*) produced elongated crystals parallel to calcite *c*‐axis.^[^ ^55^, ^58^, ^59^ ^]^ Reduced symmetry has also been observed, where the trigonal symmetry of calcite was reduced to monaxon spicule in *Kebira uteoides*.^[^ ^60^ ^]^

### 3.2 Sizes, Geometries, and Distributions of Intracrystalline Organic Materials

For biogenic calcite, ADF‐STEM electron tomography revealed the disk‐like geometries of the intra‐OMs in *A. rigida* prisms with lateral elongation along the basal plane of calcite (perpendicular to calcite *c*‐axis, Figure 4d).^[^ ^40^ ^]^ XRD measurements also confirmed such intra‐OM alignments in the calcite prisms of *Pinna nobilis* ^[^ ^53^, ^61^ ^]^ and *Atrina serrata* shells.^[^ ^43^ ^]^ These observations are consistent with our recent TEM characterizations on *A. rigida* prisms, where the geometry of individual intra‐OMs can be approximated by an equal‐axed ellipsoid with a height of ≈5 nm and a lateral span of ≈10 nm (Figure 4e,f).^[^ ^28^ ^]^ In addition, alternating regions of high‐ and low‐density of intra‐OMs were observed along the prism length.^[^ ^28^, ^40^ ^]^ At a larger scale, these intra‐OMs gradually tilts upward when approaching the prism and inter‐OM boundary (Figure 4g).^[^ ^28^ ^]^ Other TEM studies also revealed variations in terms of intra‐OM geometries and distributions. The intra‐OMs in *A. pectinata* prisms exhibit 2D elongated geometries similar to *A. rigida* prisms but with homogenous distribution, while the intra‐OMs in *P. fucata* prisms show inhomogeneous distribution and form small‐angle grain boundaries with associated crystal defects.^[^ ^50^ ^]^ In porous echinoderm calcite (*P. lividus*), on the other hand, XRD measurements indicate the parallel alignment of intra‐OMs to the calcite *c‐*axis.^[^ ^43^ ^]^ Further observations indicated that these intra‐OMs exhibited concentric layers bridged by radial threads, as revealed by the layered‐structure on the fracture surfaces (Figure 2b)^[^ ^7^, ^62^ ^]^ and the etched cross‐sections.^[^ ^63^ ^]^ Cytochemistry analysis of the skeleton‐forming cells in sea urchins revealed similar concentric layers in the mineralization organic matrix, indicating that these intra‐OMs might function as the scaffold for mineralization.^[^ ^64^ ^]^

In aragonite‐based biominerals, the intra‐OMs exhibit rather different morphologies. In the helical fibrous building blocks of *Clio pyramidata* shell, there are two types of intra‐OM geometries (Figure 4h):^[^ ^48^ ^]^ i) The diamond‐shaped inclusions (red dashed rhombus) distributed in the upper peripheral regions, and ii) the thin elongated‐sheet inclusions (blue dashed ellipse) randomly distributed over the entire cross‐section. Nacre tablets show two similar types of intra‐OMs (Figure 4i), both of which appear to be circular and randomly distributed when viewed down the (001) plane (Figure 4j).^[^ ^47^, ^65^ ^]^ However, there are depletion zones near the top and bottom surfaces of nacre tablets (yellow‐shaded bands in Figure 4k), where no intra‐OMs were detected.^[^ ^46^ ^]^ Further, other works suggested a continuous organic framework in the nacre tablets that divides the individual building blocks into nanograins.^[^ ^66^ ^]^

### 3.3 Mechanical Influence of Intracrystalline Organic Materials

Intra‐OMs modify the crystal microstructure of biominerals,^[^ ^43^ ^]^ and their mechanical influence include several aspects, such as lattice distortion, dislocation restriction (or strengthening), modification of fracture behavior through crack coalesce and deflection, and energy dissipation via the viscoelastic or plastic deformation of intra‐OMs.

The intra‐OMs typically have nanoscale dimensions (Figure 4e,f), which are 1–2 orders of magnitude larger than a crystal unit cell, for example, calcite (*a* = 4.988 Å and *c* = 17.068 Å).^[^ ^55^, ^67^ ^]^ Lattice distortions were reported in both biogenic calcite^[^ ^68^, ^69^ ^]^ and aragonite^[^ ^70^, ^71^, ^72^, ^73^ ^]^ using synchrotron‐based high‐resolution powder XRD measurements. Further, heat treatment (at temperatures above organics decomposition) resulted in relaxation of the lattice distortions, supporting the claim that the intra‐OMs contribute to lattice distortions.^[^ ^68^, ^72^ ^]^ The distortion‐resulted residual strain/stress in atomic biomineral lattice will be discussed further in Section 6.2. At a larger scale, the presence of intra‐OMs alters the degree of perfection of crystal texture, which is evidenced by decreased coherence lengths and increased anisotropy under synchrotron X‐ray radiation.^[^ ^37^, ^55^ ^]^

**Figure 5:**
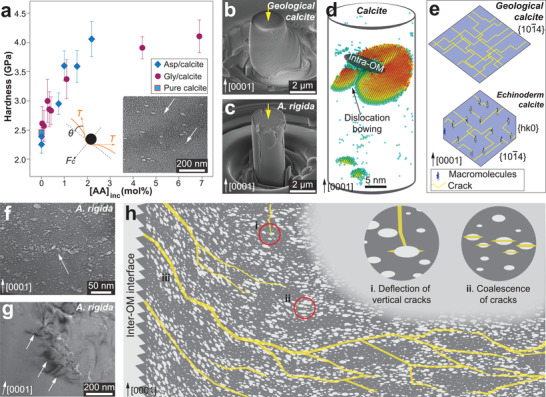
Intracrystalline organic materials: Mechanical functions. a) Hardness versus amino acid (AA) contents in synthetic calcite, where the inset shows the TEM image of synthetic calcite occluding micelles (white arrows). b,c) SEM images of b) geological and c) biogenic calcite micro‐pillars after uniaxial compression, where the geological calcite exhibits dislocation slips, and the biogenic calcite favors brittle fracture. d) Molecular dynamics (MD) simulation of *c*‐axis compression on calcite with intra‐OM, where dislocation bowing is formed around the intra‐OM. e) Schematic illustrations of crack propagation in pure calcite and biological calcite with intra‐OMs oblique to the cleavage planes.^[^ ^77^ ^]^ f,g) TEM images of biogenic calcite, showing f) the crack initiation (white arrow) through coalescence of adjacent intra‐OMs, and g) micro‐cracks (white arrows) propagating in parallel to the long axes of the intra‐OMs (perpendicular to calcite *c*‐axis [0001]). h) Regulation of crack formation and propagation in biogenic calcite by intra‐OMs, including i) crack deflection; ii) crack initiation resulted from intra‐OM coalescence; and iii) upward cracks due to the inclined defects near prism boundaries. a) Reproduced with permission.^[^ ^75^ ^]^ Copyright 2016, Springer Nature. a, inset) Reproduced with permission.^[^ ^74^ ^]^ Copyright 2011, Springer Nature. b–d,f–h) Reproduced with permission.^[^ ^28^ ^]^ Copyright 2020, Springer Nature.

The incorporation of intra‐OMs also contributes to dislocation restriction in biogenic minerals, which improves strength and hardness. A recent study by Kim et al. showed that by incorporating copolymer micelles into synthetic calcite, the composite sample did not generate cracks and parallel steps under indentation, where these features were observed on pure calcite due to aligned slip planes.^[^ ^74^ ^]^ The hardening behaviors in synthetic calcite were observed with increasing organic inclusions (**Figure** **5**a), which were explained by a dislocation‐pinning model where the organic molecules impede dislocation motion, causing it to bow out between adjacent molecules (Figure 5a, inset).^[^ ^75^ ^]^ The dislocation motion could only resume when the cutting force *F*_c_ (shearing the organic molecules) surpass the dislocation line tension *T*, and the hardness increase can be written as

(1) $$H=H_{0}+4.8\frac{F_{c}}{bL}\sqrt{\frac{F_{c}}{2T}}$$

where *H*_0_ and *H* correspond to the hardness of pure calcite and biogenic calcite with organic inclusions, respectively; *b* is the Burgers vector magnitude; and *L* is the spacing between intra‐OMs.^[^ ^75^ ^]^ The dislocation impediment mechanism by intra‐OMs has also been confirmed experimentally (Figure 5b,c) and through molecular dynamics (MD) simulations (Figure 5d).^[^ ^28^ ^]^ This mechanical strengthening effect due to the presence of intra‐OMs is similar to the precipitation hardening behavior in engineering materials, like metals and ceramics.^[^ ^76^ ^]^ The most intriguing difference is that the precipitates in engineering materials are typically harder than the matrix to resist dislocation motion, while the intra‐OMs in biogenic minerals are the softer phase.^[^ ^55^ ^]^

In addition to the strengthening effects, the intra‐OMs are also attributed to the conchoidal fracture for biogenic minerals. As discussed earlier, many studies show that intra‐OMs are absorbed onto specific crystallographic planes which may be oblique to the preferred cleavage planes.^[^ ^43^, ^61^ ^]^ For example, the intra‐OMs in echinoderm calcite are aligned along the [0001] direction (oblique to the $\left\{10\bar{1}4\right\}$ cleavage planes). It was proposed that the intra‐OMs are able to absorb and deflect the progressing cracks (Figure 5e).^[^ ^55^, ^77^ ^]^ Consistent with this hypothetic model, crack regulating behaviors from intra‐OMs were observed (Figure 5f–h):^[^ ^28^ ^]^ i) Microcracks tend to initiate at regions with high density of intra‐OMs by coalescing adjacent inclusions (Figure 5f,h‐ii); ii) the elongated geometry of individual intra‐OMs facilitated crack deflection along the horizontal direction (Figure 5g,h‐i); and iii) the upward tilting alignment of the intra‐OM inclusions guided the cracks and avoided deep fracturing into the prismatic crystal (Figure 5h‐iii). Similar crack guiding effect has also been observed in nacre tablets, where the densely clustered intra‐OMs were observed along the crack propagation pathway.^[^ ^78^ ^]^ The effects from the sizes, distribution spacing, and orientation of intra‐OMs on the fracture strength *σ*_f_ of biogenic minerals can be described quantitatively by a quasi‐brittle fracture model by considering the intra‐OMs as pre‐existing defects,

(2) $$\sigma_{f}={\left(\frac{2\pi }{11}\right)}^{1/4}L^{1/2}\left(\frac{\sigma_{Y}\sqrt{\pi a}}{20\beta (\theta )a}\right)$$

where *a* is the half length of the intra‐OMs, *L* is the spacing between intra‐OMs, *θ* is the orientation angle of intra‐OMs against the loading direction, *β*(*θ*) is a function of *θ* and frictional coefficient between the intra‐OMs and the mineral matrix, and *σ*_Y_ represents the compressive strength of pure mineral phase.^[^ ^28^ ^]^ This simplified model provides theoretical basis for the aforementioned fracture behavior observed in biogenic calcite.

Last but not least, the deformation of intra‐OMs might also contribute to energy dissipation for biominerals. However, due to their nanometer sizes, it is extremely challenging to isolate intra‐OMs for mechanical characterizations. Indirect insights may be obtained from the properties of inter‐OMs.^[^ ^79^, ^80^ ^]^ For instance, fiber pulling experiments on interlamellar fibers of freshly cleaved nacre were conducted using atomic force microscopy (AFM), which revealed the stepwise unfolding of the proteinaceous organic materials and recoverable folding.^[^ ^81^, ^82^, ^83^ ^]^ Tension and relaxation tests on the intercrystalline organic framework from nacre (by demineralization) also revealed a time‐dependent viscoelastic behavior.^[^ ^84^ ^]^ On the other hand, from the deformation of individual nacre tablets, nanograin rotation, and deformation were revealed under mechanical loadings.^[^ ^85^, ^86^, ^87^ ^]^ Such deformation mechanisms are expected to be facilitated by the viscoelastic or plastic deformation of the continuous intra‐OM framework between the nanograins.^[^ ^88^ ^]^

## 4 Trace Elements (Inorganic Impurities)

In this section, we discuss the trace elements in biominerals. Here the elements other than the main constituent elements of minerals are considered as trace elements. For instance, in a calcium carbonate‐based mineral matrix, elements other than Ca, C, and O are the trace elements. The trace elements within intra‐OMs are not considered here. As such, the trace elements in the biomineral matrix can also be referred to as inorganic impurities, in correspondence to the organic inclusions discussed in Section 3. The inorganic impurities exist in the mineral matrix via two forms: Direct substitution in the crystal lattice (e.g., Mg^2+^ substituting Ca^2+^ in calcite,^[^ ^89^ ^]^ and F^−^ substituting OH^−^ in HAP^[^ ^90^, ^91^ ^]^), and nanoscale aggregates with high‐density of element substitutions (e.g., Mg‐rich nano‐domains in brittle star *Ophiocoma wendtii* ^[^ ^24^, ^92^ ^]^). In this section, we focus on the direct substitution, while the inorganic nano‐domains will be introduced in Section 6.3 as a special type of structural form for inducing residual stresses.

### 4.1 Magnesium in Calcium Carbonates

**Figure 6:**
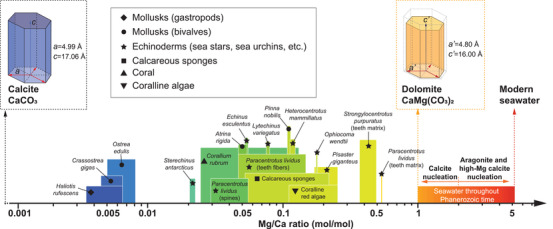
Magnesium contents in different calcite‐based biominerals. The log‐scale Mg/Ca ratios are plotted on the horizontal axis, where the widths of the bars indicate the ranges of Mg in corresponding biomineral systems, and the height variations do not indicate anything. Several milestone values are marked, including Mg/Ca = 0, 1, and 5.2, which indicate corresponding ratios in pure calcite, dolomite, and modern seawater, respectively. As Mg content increases, the lattice parameters are expected to decrease, as evidenced by the comparison between pure calcite (*a* = 4.99 Å, *c* = 17.06 Å) and dolomite (*a* = 4.80 Å, *a* = 16.00 Å). The Mg concentration data in biogenic calcites are obtained from refs.[24, 25, 68, 95, 96, 97, 103]

In abiotic minerals, Sr and Mg are the most common substitutes for Ca, for both carbonate and apatite crystals.^[^ ^89^, ^93^, ^94^ ^]^ Similarly, Mg is also commonly found in biominerals, especially in biogenic calcite, for example, sea urchin spines^[^ ^95^, ^96^ ^]^ and teeth,^[^ ^97^, ^98^ ^]^ prisms in mollusk shells,^[^ ^68^, ^99^ ^]^ brittle stars.^[^ ^100^ ^]^ This could result from the natural marine environment, where the Mg/Ca ratio of sea water has varied between 1.0 and 5.2 throughout the Phanerozoic Eon,^[^ ^101^ ^]^ and a higher Mg/Ca ratio (>2) favors nucleation of high‐Mg calcite or aragonite.^[^ ^102^ ^]^ **Figure** **6** summarizes the Mg/Ca ratios (mol/mol) in reported biogenic calcite systems.^[^ ^24^, ^25^, ^68^, ^95^, ^96^, ^97^, ^103^ ^]^ This graph indicates that the Mg concentration can vary significantly among species (Mg/Ca ratio between ≈0.005 and 0.5), where the echinoderm skeletons typically reside toward the high end.^[^ ^96^ ^]^ Several landmark values should be noted, where Mg/Ca (mol/mol) = 0, 1, and 5.2 represent pure calcite CaCO_3_, dolomite CaMg(CO_3_)_2_, and modern seawater level, respectively. On the other hand, Mg doping in aragonite is less favored,^[^ ^93^ ^]^ as Mg^2+^, if substituted for Ca^2+^ in aragonite, would have unstable ninefold coordination, which prevents Mg^2+^ from entering the bulk aragonite.^[^ ^104^ ^]^ Therefore, biogenic aragonite typically has a much lower Mg concentration than calcite, for example, ≈25 000 ppm Mg in calcite prisms versus 2000 ppm in aragonite nacre from a bivalve mollusk *Pinctada margaritifera*.^[^ ^105^ ^]^ In addition, based on X‐ray absorption near edge spectroscopy of the aragonite prisms in *Arctica islandica*, Mg in biogenic aragonite was hosted by a disordered phase (e.g., organic components), rather than direct substitution into aragonite lattice.^[^ ^106^ ^]^

#### 4.1.1 Effect of Mg on Biomineralization

**Figure 7:**
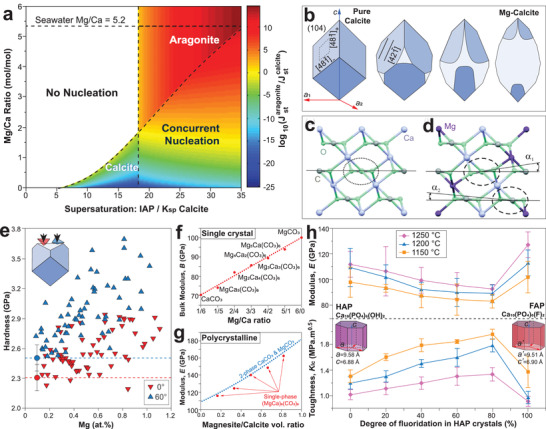
Mechanical influence of trace‐element substitution in biominerals, including a–g) Mg in calcite and h) F in hydroxyapatite (HAP). a) Kinetic phase diagram of the relative nucleation rates between aragonite and calcite in relation to the Mg/Ca ratio and supersaturation level. b) Schematic model for the effect of Mg on calcite morphology, that is, from rhombohedral pure form to an elongated form of Mg‐containing crystal form.^[^ ^89^ ^]^ c,d) Atomic structure of c) calcite CaCO_3_ with in‐plane oriented ${\mathrm{CO}}_{3}^{2-}$groups (dotted oval) and d) CaMg(CO_3_)_2_ with locally tilted ${\mathrm{CO}}_{3}^{2-}$ groups. e) Nanoindentation hardness as a function of Mg content in synthetic calcite, where the downward triangles (red filled) and upward triangles (blue filled) represent indentations at 0° and 60° azimuthal angles, respectively. Correspondingly colored circles and dashed lines refer to the hardness of pure calcite. f,g) DFT results on f) the bulk modulus of single‐crystal Mg‐calcite versus increasing Mg/Ca ratio, and g) elastic modulus of a polycrystalline mixture of calcite and magnesite (blue lines) and single‐phase Mg‐calcite (red points). h) Indentation modulus and fracture toughness of sintered F‐containing HAP crystals versus increasing fluoridation at different temperatures. a) Reproduced with permission.^[^ ^102^ ^]^ Copyright 2015, National Academy of Sciences, U.S.A. c,d,f) Reproduced with permission.^[^ ^116^ ^]^ Copyright 2010, Elsevier. e) Reproduced with permission.^[^ ^99^ ^]^ Copyright 2012, Springer Nature. g) Reproduced with permission.^[^ ^121^ ^]^ Copyright 2013, Elsevier. h) Reproduced with permission.^[^ ^91^ ^]^ Copyright 2004, Elsevier.

The Mg/Ca ratio has a profound influence on CaCO_3_ polymorph selection. At ambient conditions, aragonite is a metastable polymorph of CaCO_3_, while calcite is the stable phase.^[^ ^102^ ^]^ However, metastable polymorphs can be stabilized at the nanoscale if they have lower surface energy than the stable phase.^[^ ^107^, ^108^ ^]^ The Mg/Ca ratio, aside from the temperature, saturation state, and salinity in marine environment, controls the CaCO_3_ polymorph selection by affecting the surface energy of the solvent‐crystal interface for different polymorphs.^[^ ^101^, ^102^ ^]^ **Figure** **7**a plots the kinetic phase diagram of competitive nucleation rates between aragonite and calcite as a function of Mg/Ca ratio and supersaturation (*σ*).^[^ ^102^ ^]^ Calcite nucleation starts at a low supersaturation of *σ*_calcite_ = 5; as the Mg/Ca ratio increases, the calcite nucleation is inhibited and only occurs at higher supersaturation.^[^ ^102^, ^109^ ^]^ On the other hand, aragonite nucleation dominates at *σ*_calcite_ ≥ 18 and is not influenced by Mg/Ca ratios. Therefore, the Mg/Ca ratio of ≈2 marks an effective boundary for polymorph selection between calcite and aragonite, where similar nucleation rates for the two polymorphs are observed (concurrent nucleation, Figure 7a).^[^ ^102^ ^]^

Apart from polymorph control, the presence of Mg also induces a morphological change of the calcite crystals. For example, in situ AFM observations revealed that the rhombohedral shaped calcite crystals formed new [42$\bar{1}$] step directions, which gradually developed into an elongated form with the presence of Mg (Figure 7b).^[^ ^89^ ^]^ In addition, calcite growth on carboxylic acid monolayers revealed that Mg induced i) more uniform $\left\{10\bar{1}4\right\}$ rhombohedral nucleation from the (012) planes at low Mg^2+^ concentrations (Mg/Ca = 0–0.5), ii) elongated crystals along the *c*‐axis with a rough texture at Mg/Ca > 0.5, and iii) needle‐ or seed‐like crystals (further elongation along the *c*‐axis) at Mg/Ca > 1.5.^[^ ^110^ ^]^ It is well known that morphological modifications of calcite crystals can also be achieved by the addition of organic substances, such as, peptides^[^ ^111^ ^]^ and extracted proteins.^[^ ^55^, ^58^, ^59^ ^]^

Mg has also been reported to stabilize the amorphous calcium carbonate (ACC), the precursor phase in many biomineralization processes.^[^ ^112^ ^]^ In vitro biomimetic synthesis indicated that acidic polypeptides induced ACC formation through a polymer‐induced liquid‐precursor process, and Mg^2+^ reduces the amount of acidic polypeptides needed for stabilizing ACC.^[^ ^109^ ^]^ Similar in vitro experiments using macromolecules extracted from sea urchin 48‐hour larval spicules (*Strongylocentrotus purpuratus*, > 50% ACC) also showed that the ACC phase could only be produced with the presence of Mg^2+^.^[^ ^113^ ^]^ To understand the stabilizing mechanism, the chemical environment around Mg in ACCs and crystalline calcite was studied using X‐ray absorption spectra; the analyzed results showed that the compact structure around Mg (shorter Mg—O bond lengths induces distortion in the CaCO_3_ host mineral and inhibits its crystallization.^[^ ^114^ ^]^ Another interesting example of Mg‐doped ACC in biomineralization is the echinoderm calcite in brittle star *O. wendtii*: It was proposed that Mg‐ACC underwent a spinodal decomposition into Mg‐rich nanodomains (≈40 mol%) and Mg‐depleted calcite matrix (≈13 mol%) before crystallization.^[^ ^100^ ^]^

#### 4.1.2 Effect of Mg on Mechanical Properties

In terms of mechanical effects, Mg substitution in calcite causes lattice distortion at the atomic scale. An early study on sea urchin spines and test plates revealed some weak XRD peaks (0003 and 0009 reflections, etc.), which were supposed to be forbidden in perfect calcite (*R* $\bar{3}$ *c* space group).^[^ ^115^ ^]^ It was proposed that those reflections could result from the local superstructure domains with periodic Mg‐calcite layering (overall Ca:Mg = 3:1, alternating Ca‐Ca and Ca‐Mg layers along *c*‐axis).^[^ ^115^ ^]^ However, there is currently no direct experimental evidence showing such periodic substitution of Mg in biogenic calcite. At atomic scale of Mg‐calcite, computational studies based on density functional theory (DFT) showed that Mg substitution induced off‐planar tilting of CO_3_^2−^ group at a balanced state (Figure 7c,d).^[^ ^116^ ^]^ Considering Mg^2+^ is smaller than Ca^2+^, the Mg‐calcite lattice should also shrink. This can be inferred from the comparison of the lattice parameters between pure calcite (CaCO_3_, *a* = 4.988 Å, *c* = 17.068 Å) and magnesite (MgCO_3_, *a* = 4.632 Å, *c* = 15.007 Å).^[^ ^67^ ^]^ Synchrotron X‐ray studies further revealed a linear correlation between the lattice parameters of Mg‐calcite with Mg concentrations,^[^ ^68^ ^]^

(3) $$\left\{\begin{array}{c} a=-0.003537x+4.985720\\ c=-0.020560x+17.06634 \end{array}\right.$$

where *x* defines the Mg content by at.%, and the coefficients vary slightly in different studies.^[^ ^117^, ^118^ ^]^ The lattice distortion caused by the trace element substitution enhances the material in a way similar to solute strengthening in alloying metals, in which the solute atoms (point defects) generate stress fields due to lattice mismatch and impede dislocation motion.^[^ ^119^ ^]^ Of special attention is that the lattice distortion induced by Mg is negative, that is, inducing lattice shrinkage, while the intra‐OMs induce lattice expansion (see Section 3).^[^ ^68^ ^]^

Nanoindentation measurements show that the incorporation of Mg increases the hardness and modulus in both biological^[^ ^23^, ^25^, ^98^ ^]^ and synthetic calcite (Figure 7e).^[^ ^99^, ^120^ ^]^ It is estimated that the Mg content accounts for 20–50% of the hardness increase in biogenic calcite.^[^ ^23^, ^99^ ^]^ In addition, the ab initio DFT simulations revealed that the bulk modulus of single‐crystal Mg‐calcite is linearly dependent on the Mg concentration (Figure 7f).^[^ ^116^, ^121^ ^]^ It also revealed that the transformation of Mg‐calcite as a solid solution to a two‐phase composite (a mixture of calcite and magnesite) is neither thermodynamically favorable nor significantly beneficial for mechanical stiffening (Figure 7g).^[^ ^121^ ^]^ This conclusion is supported by XRD measurements where Mg‐containing calcites diffract as single‐phase crystals.^[^ ^96^, ^122^ ^]^

### 4.2 Fluoride in Hydroxyapatites

Another common example of element substitution in biominerals is the fluoridation of HAP crystals, that is, the substitution of the OH^−^ groups with F^−^ in Ca_10_(PO4)_6_(OH)_2_. F‐HAP crystals are commonly used as the basic building blocks in the teeth of many fish (e.g., sharks,^[^ ^90^, ^123^ ^]^ parrotfish,^[^ ^124^ ^]^ and black drum^[^ ^125^ ^]^) and the impact surface of the stomatopod dactyl clubs.^[^ ^126^, ^127^ ^]^ F substitution in HAP also induces change in lattice parameters (shrinkage in *a*‐axis while expansion along *c*‐axis).^[^ ^91^, ^128^, ^129^ ^]^ The stiffening and hardening effect of F deposition has also been characterized by comparing the moduli^[^ ^128^ ^]^ and indentation hardness^[^ ^90^ ^]^ of HAP and fluorapatite crystals. In polycrystalline HAP, however, the mechanical contribution of fluoridation is more complicated. Based on the nanoindentation results on the compressed and sintered samples prepared from F‐HAP powders, studies showed non‐monotonic change in mechanical properties with increasing fluoridation.^[^ ^129^ ^]^ As shown in Figure 7h, the minimum modulus and the maximum fracture toughness was achieved at 80% fluoridation, that is, Ca_10_(PO_4_)_6_(OH)_0.4_F_1.6_.^[^ ^91^ ^]^ Therefore, the F‐HAP mineral phase in fish teeth might contribute to an optimal toughness.^[^ ^90^ ^]^

### 4.3 Chemical Gradients in Biominerals and Biomineral Composites

Apart from the local substitutions, large‐scale gradient distributions of trace elements in biomineral composites have also been observed. At composite level, the chemical gradients are usually associated with structural gradients of the mineral building blocks (e.g., arrangements, distribution, dimensions, and orientations), which work in synergy for specific functional requirements, including structural support, contact damage resistance, and interfacial enhancement.^[^ ^130^ ^]^ For example, the multi‐layered exoskeleton of the crab *Cancer pagurus* has selective distributions of trace elements, with Mg and P concentrated in the exocuticle, constructing a load‐bearing outer layer.^[^ ^116^ ^]^ Another example is the raptorial appendages of mantis shrimps, including the smashing club of *Odontodactylus scyllarus* ^[^ ^126^ ^]^ and the spearing club of *Lysiosquilla*.^[^ ^127^ ^]^ In these structures, the selective gradient depositions of the trace elements (Mg, F, and S) correlate well with the property gradients on the cross sections. In contrast, large‐scale chemical gradients are less recognized at the level of individual biomineral building blocks. The calcite prisms in *P. nobilis* shells exhibit layered distributions of S and Mg due to the layered growth, where S is associated with the organics and Mg is correlated the trace elements in calcite lattice.^[^ ^131^ ^]^ Such chemical gradients in biominerals add another structural hierarchy regarding the functional designs.

## 5 Crystalline Features

**Figure 8:**
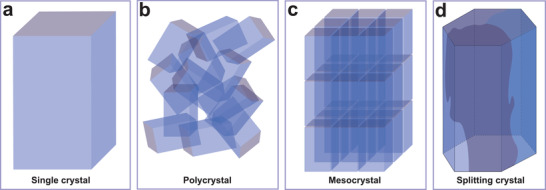
Schematic representations of the typical crystalline features. a) Single crystal with a continuous mineral matrix. b) Polycrystal with random crystallographic alignment. c) Mesocrystal with aligned crystallographic orientation and internal porosity. d) Splitting crystal found in biogenic calcite prisms, where the subdomains are formed by slightly crystallographic rotations.

It is commonly assumed that the individual biomineral building blocks are single crystals. However, continuing experimental evidence suggests that there are varieties of crystalline features in biominerals. Here, we introduce and discuss the amorphous (non‐crystalline) solid and four types of crystalline features in biominerals (**Figure** **8**), including single crystal, polycrystal, mesocrystal,^[^ ^132^, ^133^ ^]^ and a special example of splitting crystal found in biogenic calcite prisms.^[^ ^134^, ^135^ ^]^ Amorphous solids refer to the non‐crystalline structure without a periodic arrangement of the constituent atoms/ions. Single crystal is a continuous crystalline structure with no grain boundary, which may be formed via classical ion‐by‐ion attachment or non‐classical pathways (Figure 8a).^[^ ^136^ ^]^ Polycrystal is the aggregation of nanocrystals either with or without crystallographic alignment among grains (Figure 8b). A mesocrystal can be considered as a self‐assembled superstructure of numerous nanocrystals aligned in the same crystallographic orientation (Figure 8c).^[^ ^137^ ^]^ In addition, the splitting crystal is characterized by the presence of sub‐domains with slight crystallographic misorientations (Figure 8d).^[^ ^134^, ^135^ ^]^ In this section, these crystalline features in calcium carbonate biominerals and corresponding mechanical effects are introduced in the following order: i) Mesocrystal, ii) amorphous phase ACC, and iii) comparison of single crystal, polycrystal, splitting crystal, and nanogranular textures.

### 5.1 Mesocrystal

**Figure 9:**
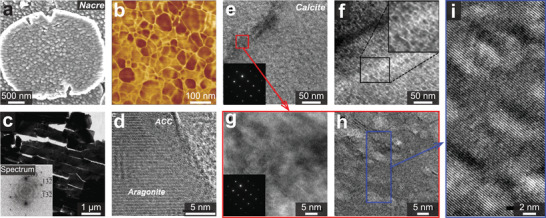
Mesocrystal features in calcium carbonate biominerals. a) SEM image of a protease treated nacre tablet from *Pteria hirundo* shell revealing nanoparticle‐like morphology. b) AFM image in phase contrast mode showing the intracrystalline organic matrix in nacre tablet (*P. maxima*). c,d) TEM micrographs of nacre platelets showing c) a thin cut across nacre thickness (*Haliotis laevigata*), and d) HRTEM micrograph of a selected crystalline aragonite tablet with constant lattice fringe covered by an ACC layer. e–i) TEM images of a synthetic calcite/PSS‐MA crystal. TEM images taken at the same area e) at focus showing a continuous mineral matrix, and f) under focus showing a nanogranular structure. Higher magnification TEM images of the area boxed in (e), showing the same area g) before and h) after beam damage. i) A HRTEM image revealing perfect lattice continuity even after beam damage. a) Reproduced with permission.^[^ ^139^ ^]^ Copyright 2013, Elsevier. b) Reproduced with permission.^[^ ^66^ ^]^ Copyright 2005, Elsevier. c,d) Reproduced with permission.^[^ ^170^ ^]^ Copyright 2005, National Academy of Sciences, U.S.A. e–i) Reproduced with permission.^[^ ^160^ ^]^ Copyright 2014, Springer Nature.

First of all, many biominerals have been reported to consist of nanograins in their building blocks, for example, nacre tablets,^[^ ^138^, ^139^, ^140^ ^]^ echinoderm calcite,^[^ ^6^, ^7^, ^141^ ^]^ and prismatic calcite,^[^ ^142^ ^]^ yet these biominerals diffract as single crystals. To describe such structural morphologies formed through non‐classical crystallization pathways, Cölfen et al. proposed the concept “mesocrystal” in 2005.^[^ ^143^, ^144^ ^]^ Based on the definition then, mesocrystals are made up of individual crystalline nanoparticles arranged with the same crystallographic orientation via mesoscale aggregation and alignment.^[^ ^145^, ^146^ ^]^ Since then, the concept of mesocrystal has received great attention, not limited to biominerals but also synthetic materials.^[^ ^143^, ^147^, ^148^, ^149^ ^]^ As shown in **Figure** **9**a–d, most studies classified biominerals as mesocrystals based on their nanogranular morphologies and co‐aligned crystallographic orientations, which are supported by high‐resolution imaging (e.g., SEM, TEM, AFM) and diffraction experiments (e.g., XRD, SEAD).

The concept of “mesocrystal” for biominerals have also been evolving in the past decade. More studies recently reported that ACC is the precursor phase for the biomineralization process (not crystalline nanoparticles).^[^ ^150^, ^151^, ^152^, ^153^ ^]^ In addition, high internal surface area is expected in mesocrystals due to the unfilled interstices between nanoparticles.^[^ ^154^ ^]^ In biominerals such as sea urchin spines, however, the surface areas measured by Brunauer–Emmett–Teller method were two orders lower than synthetic mesocrystals, which disputed the claim of mesocrystal in sea urchin spines.^[^ ^155^ ^]^ Considering these new understanding of biomineral structure and formation, Song and Cölfen further expanded the concept of “mesocrystal” to include structures formed by aggregation of amorphous nanoparticles in 2010.^[^ ^137^ ^]^ In addition, Zhou and O'Brien suggested that the sole criterion to determine whether a material is mesocrystal should be its unique crystallographically hierarchical structure, not the formation mechanism or the level of porosity, as the pores could be occupied by organic/inorganic substances.^[^ ^156^ ^]^ Different formation mechanisms of mesocrystals are possible, including nanoparticle alignment by an organic matrix, physical fields/forces (e.g., electric, magnetic, dipole fields), mineral bridges connecting adjacent nanoparticles,^[^ ^157^ ^]^ space constraints and self‐similar growth, topotactic reactions, etc.^[^ ^137^, ^158^ ^]^ With the updated concept, it is now generally accepted that biominerals with crystallographically co‐aligned nanograins are considered as mesocrystals.^[^ ^159^ ^]^

However, great care should be taken when classifying minerals as mesocrystals. As reported by Meldrum et al., different TEM imaging conditions may lead to completely different interpretations of the ultrastructure, for example, continuous matrix with lattice continuity at focus versus an apparent nanoparticulate structure in the same area when under focus (Figure 9e–i).^[^ ^160^ ^]^

### 5.2 Amorphous Calcium Carbonate

**Figure 10:**
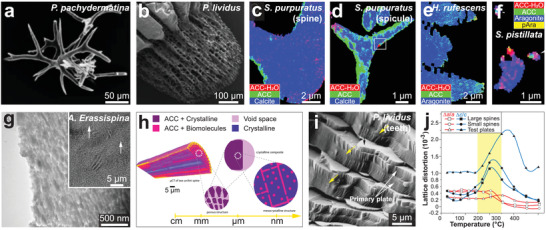
Amorphous calcium carbonate (ACC) in biominerals and its mechanical contribution. a) SEM image of body spicules from *P. pachydermatina*, composed of stable ACC. b) SEM image of the regenerated sea urchin spine of *P. lividus*, where the ACC presented as transient precursors during biomineralization. c–f) Component maps of forming c,d) biogenic calcite and e,f) biogenic aragonite measured by X‐ray photoEmission electron spectroMicroscopy (X‐PEEM), including c) regenerating spine and d) larval spicules from sea urchin *S. purpuratus*, e) fresh nacre tablets in red abalone shell *Haliotis rufescens*, and f) mineralizing particles in tissue of coral *S. pistillata*. g) TEM image of a thin section from sea urchin spine *Authoeidaris Erassispina* showing nanoparticle ultrastructure, where the inset shows defect regions in lattice fringe (white arrows). h) Schematic illustration of structural hierarchy in sea urchin spine, which is composed of ordered nanocrystal building units (purple) with ACC inclusions and surface layers (pink). i) Fracture surface of primary plates in sea urchin teeth (*P. lividus*), revealing the ACC layer (yellow arrows) sandwiched in the single‐crystal calcite primary plates (white arrows). j) Temperature dependence of lattice distortions (∆*a*/*a* and ∆*c*/*c*) for large spines (squares), small spines (circles), and test plates (triangles) of *P. lividus* sea urchin. a) Reproduced with permission.^[^ ^150^ ^]^ Copyright 2003, John Wiley and Sons. b) Reproduced with permission.^[^ ^151^ ^]^ Copyright 2004, AAAS. c) Reproduced with permission.^[^ ^174^ ^]^ Copyright 2019, Elsevier. d) Reproduced with permission.^[^ ^173^ ^]^ Copyright 2008, National Academy of Sciences, U.S.A. e) Reproduced with permission.^[^ ^171^ ^]^ Copyright 2015, American Chemical Society. f) Reproduced with permission.^[^ ^172^ ^]^ Copyright 2017, National Academy of Sciences, U.S.A. g,h) Reproduced with permission.^[^ ^141^ ^]^ Copyright 2012, National Academy of Sciences, U.S.A. i) Reproduced with permission.^[^ ^97^ ^]^ Copyright 1997, Royal Society. j) Reproduced with permission.^[^ ^185^ ^]^ Copyright 2018, American Chemical Society.

ACC exists in biominerals either as a stable form that functions as structural components or as transient precursors to form the crystallized phases during biomineralization.^[^ ^150^ ^]^ The former stable type has been reported in several systems, including the body spicules of sea tulip *Pyura pachydermatina* (**Figure** **10**a),^[^ ^150^ ^]^ calcareous sponge *Clathrina contorta*,^[^ ^60^, ^161^ ^]^ and cystoliths in the leaves of *Ficus microcarpa*.^[^ ^162^, ^163^ ^]^ In addition, stable ACC is also widely used for structural purposes in crustaceans (crabs, lobsters, etc.), which also takes advantage of ACC's high solubility for periodic molting.^[^ ^150^, ^164^ ^]^ In terms of mechanical properties, ACC has the advantage of being isotropic and less brittle compared to the crystalline phases.^[^ ^161^ ^]^ An experimental study comparing the nanoindentation properties of synthetic ACC and calcite in thin films showed that ACC had an averaged hardness of 0.75 GPa and modulus of 25–30 GPa, significantly lower than calcite (hardness of 5–5.5 GPa, and modulus of 59–60 GPa).^[^ ^165^ ^]^ Systematic studies of the mechanical contribution of ACC in biomineral systems are still lacking.

As precursors, ACC is not stable and can transform to calcite or aragonite.^[^ ^166^ ^]^ The ACC‐to‐calcite transition was first revealed in sea urchin larva spicules,^[^ ^167^, ^168^ ^]^ and later found during the regeneration of adult spines as well (Figure 10b).^[^ ^151^ ^]^ ACC also exists in aragonite‐based biominerals, including bivalve larval shells,^[^ ^169^ ^]^ developing nacre in gastropod shells,^[^ ^170^, ^171^ ^]^ and coral skeletons.^[^ ^172^ ^]^ Recently, the mineralization pathway for ACC in echinoderm calcite was shown to follow a stepwise sequence: Hydrated ACC (ACC•H_2_O) → dehydrated ACC → calcite (Figure 10c,d).^[^ ^152^, ^173^, ^174^ ^]^ The ACC‐to‐aragonite transitions showed variations in different systems: the mineralization of nacre tablets follows a similar path to biogenic calcite, that is, ACC•H_2_O → dehydrated ACC → aragonite (Figure 10e),^[^ ^171^ ^]^ whereas coral mineralization exhibits an additional phase, that is, poorly crystalline aragonite (pAra) stored in the living tissue and then delivered to the mineralization front (Figure 10f).^[^ ^172^ ^]^ There have been increasing studies using MD simulations to investigate the local structures of ACC (ordering, short‐range structures, near‐neighbor coordination, etc.)^[^ ^175^, ^176^, ^177^, ^178^, ^179^ ^]^ and phase transitions (to calcite and aragonite).^[^ ^180^, ^181^, ^182^, ^183^, ^184^ ^]^ In addition, ACC was also detected in some mature biominerals, which could result from incomplete crystallization.^[^ ^185^ ^]^ For example, ≈8 at% ACC is distributed in the adult spines of sea urchin *A. Erassispina* (Figure 10g), where the ACC exists in the form of inclusions and interfaces (pink color, Figure 10h).^[^ ^141^ ^]^ Another example of ACC identified in mature biominerals is sea urchin teeth, where the ACC (yellow arrows in Figure 10i) is sandwiched between single‐crystal primary plates (white arrows in Figure 10i).^[^ ^97^ ^]^

The crystallization of ACC has a profound influence on the structural and mechanical properties of biominerals. First, significant lattice expansion was detected when echinoderm calcite was heated to 200 °C, corresponding to heat‐assisted ACC‐to‐calcite crystallization (Figure 10j).^[^ ^185^ ^]^ It is assumed that ACC‐to‐calcite crystallization is accompanied by the reduction of specific volume per molecule, thus the forces acting at organic/inorganic interfaces work against the shrinkage, producing tensile strains in the forming lattice.^[^ ^185^, ^186^ ^]^ The influence of the associated residual strains will be discussed in more detail in Section 6.2. In addition, ACC in crystalline minerals contributes to crack deflection at the ACC/crystal boundary, as evidenced by the drastic change of fracture characteristics across the primary plates in sea urchin teeth (Figure 10i).^[^ ^97^ ^]^

### 5.3 Single Crystal, Polycrystal, Splitting Crystal, versus Nanograins

**Figure 11:**
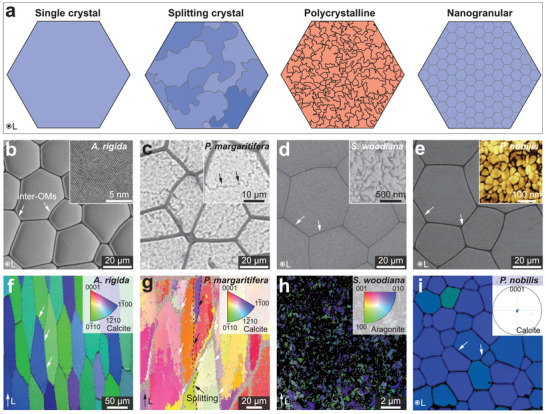
Comparison of single crystal, splitting crystal, polycrystal, and nanogranular features in prismatic biominerals. a) Schematics of crystalline features in prismatic building blocks from seashells, including single crystal (monocrystalline), splitting crystal, polycrystal, and nanogranular features (mesocrystal). b–e) SEM images of cross sections perpendicular to the length axis (“L”) of the prisms, corresponding to the four types of crystalline features, that is, b) single crystal *A. rigida*, c) splitting crystal *P. margaritifera*, d) polycrystal *S. woodiana*, and e) nanogranular *P. nobilis*. The inset of (b) show constant lattice fringe in *A. rigida* prism, the insets of (c) and (d) magnify corresponding crystalline features, and the inset of (d) show the AFM image *P. nobilis* revealing its nanogranular textures. f–i) EBSD images on the four prisms in (b–e). The intercrystalline organics (inter‐OMs) are marked with white arrows, while the boundaries between the splitting crystal are marked with black arrows and dashed lines in (g). b,f,d,h) Reproduced with permission.^[^ ^35^ ^]^ Copyright 2021, American Chemical Society. c) Reproduced with permission.^[^ ^187^ ^]^ Copyright 2016, Royal Society. e) Reproduced with permission.^[^ ^190^ ^]^ Copyright 2016, John Wiley and Sons. e, inset) Reproduced with permission.^[^ ^142^ ^]^ Copyright 2012, Royal Society of Chemistry. g) Reproduced with permission.^[^ ^135^ ^]^ Copyright 2013, Royal Society. i) Reproduced with permission.^[^ ^189^ ^]^ Copyright 2019, Acta Biomaterialia.

In Section 5.1, we introduced the concept of mesocrystals to refer the nanogranular morphology of many biominerals while still diffracting as single crystals. Here additional variations in crystallographic characteristics within individual biomineral building blocks are discussed, including single crystal, splitting crystal, polycrystals, and nanogranular features (or mesocrystal, **Figure** **11**a). The prismatic crystals in mollusk shells represent excellent models for direct comparison of these distinct crystalline features, as shown by the SEM (Figure 11b–e) and electron back‐scattered diffraction (EBSD) images (Figure 11f–i), that is, the single‐crystal calcite in *A. rigida*,^[^ ^28^, ^35^ ^]^ the splitting‐crystal calcite in *P. margaritifera*,^[^ ^135^, ^187^, ^188^ ^]^ the polycrystalline aragonite in *S. woodiana*,^[^ ^35^ ^]^ and the nanogranular calcite in *P. nobilis*.^[^ ^142^, ^189^, ^190^ ^]^ First, comparing the *A. rigida* and *P. nobilis* prisms, the single‐crystal prism has a continuous mineral matrix (Figure 11b), while the mineral matrix in the nanogranular crystal is interrupted by intergranular organics (a form of intra‐OMs, Figure 11e),^[^ ^142^ ^]^ even though they both have uniform crystallographic orientation in individual prisms (Figure 11f,i). Some high‐resolution TEM studies revealed similar nanogranular morphologies in single‐crystal biominerals and characterized them as mesocrystals. Second, the polycrystal has similar grain‐like particles to nanogranular structure (Figure 11d), but with larger grain sizes (up to ≈500 nm) with clear crystallographic misorientations (Figure 11h). Third, the splitting crystal is only reported in calcite prisms of several mollusks, which differs from polycrystal by having much larger and irregular domains (up to tens of micrometers in the largest dimension) with no organic interfaces between the splitting domains (black arrows and dashed lines in Figure 11c,g).^[^ ^191^ ^]^ Interestingly, the splitting crystal often starts as a single unit with sporadic crystallographic orientations (up to 30° tilt between the calcite *c*‐axis and the direction of growth), and then split into subdomains with subtle changes in the crystallographic orientations.^[^ ^134^, ^135^, ^192^ ^]^ For example, the crystal splitting in *P. fucata* prisms is produced by rotation about the calcite *c*‐axis (also the axial direction of prisms),^[^ ^134^ ^]^ while *P. margaritifera* and *Pinctada nigra* prisms have predominant growth perpendicular to the calcite *c*‐axis.^[^ ^135^, ^191^, ^192^ ^]^

**Figure 12:**
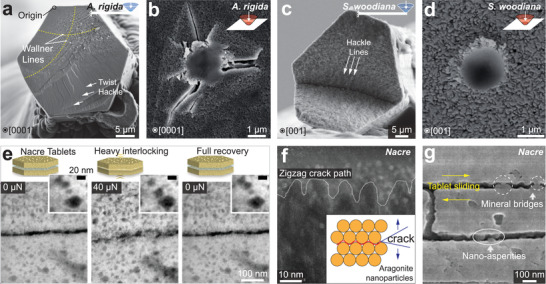
Mechanical comparison of a,b) single‐crystal, c,d) polycrystal, and e–g) nanogranular crystalline features in biogenic CaCO_3_ minerals. a–d) SEM images of the fracture patterns in (a,b) single‐crystal *A. rigida* prisms and c,d) polycrystalline *S. woodiana* prisms under a,c) mico‐bending and b,d) nanoindentation. e) High‐resolution STEM image series of two nacre tablets before compression, under 40 µN compressive load, and full recovery after removing load. Insets focus on the movement of intra‐OMs showing the deformation and complete recovery of the tablets. f) Dark‐field TEM image of nacre showing the wavy crack propagation at the nanometer scale, where the insect schematic shows the advancing crack propagating along the boundaries of aragonite nanoparticles. g) SEM image of nacre tablets and the mineral bridges (dashed white circles) and nano‐asperities (white oval) at the lamellae boundaries. a–d) Reproduced with permission.^[^ ^35^ ^]^ Copyright 2021, American Chemical Society. e) Reproduced with permission.^[^ ^199^ ^]^ Copyright 2019, Springer Nature. f) Reproduced with permission.^[^ ^200^ ^]^ Copyright 2013, Springer Nature. g) Reproduced with permission.^[^ ^201^ ^]^ Copyright 2001, Springer Nature.

The different crystalline features directly contribute to their different behaviors of biominerals under loading. Our recent work provided a comparison of the single‐crystal and polycrystalline prisms under micro‐bending (**Figure** **12**a,c) and nanoindentation conditions (Figure 12b,d).^[^ ^35^ ^]^ In single‐crystal prisms (*A. rigida*), micro‐bending tests yielded similar moduli to the results obtained by micropillar compression, because the continuous mineral matrix dominates the elastic properties.^[^ ^28^ ^]^ The fracture surfaces exhibited glass‐like conchoidal features due to the incorporated intra‐OMs (Figure 12a. Also see Section 3.3). By contrast, the polycrystalline prisms (*S. woodiana*) exhibited the characteristic intergranular fracture morphology due to the weak GBs in between adjacent grains (Figure 12c). Under concentrated indentation loadings, the single‐crystal calcite matrix in *A. rigida* favored threefold cleavage fracture along $\left\{10\bar{1}4\right\}$ planes (Figure 12b), while the polycrystalline aragonite in *S. woodiana* showed superior damage localization by deflecting cracks between GBs (Figure 12d). In comparison, the crystal‐splitting prims may represent a gradient design of crystal textures, where a greater spread of lattice titling correlates positively with higher Vickers microindentation hardness^[^ ^188^ ^]^ and wear resistance.^[^ ^193^ ^]^

In addition to prismatic calcite, sea urchin teeth represent another example with multiple crystalline features, where the polycrystalline calcite matrix show higher modulus, hardness, and crack resistance than the single‐crystal calcite fibers and plates; higher concentrations of Mg in the polycrystalline matrix also contributed to the property enhancement.^[^ ^98^ ^]^ Coral skeleton provides another interesting example of polycrystalline biogenic aragonite, where the radial growth (along *c*‐axes) of crystal bundles develops feather‐like spherulites, and therefore crystallographic misorientations can be readily observed from adjacent crystals.^[^ ^194^ ^]^ Such spherulite morphology leads to isotropic indentation hardness and effective modulus regardless of the anisotropic aragonite crystal.^[^ ^195^ ^]^

Regarding the nanogranular features in biominerals, comprehensive reviews can in found in refs. [196, 197]. Gao et al. proposed a theoretical basis for understanding the mechanical advantage of nanograins: The nanograins become insensitive to intrinsic flaws when smaller than a critical size and can thus approach the theoretical strength.^[^ ^198^ ^]^ As for experimental observations, there have been extensive studies on the nanogranular biogenic aragonite, especially nacre. Many strengthening and toughening mechanisms has been proposed in the literature, including recoverable deformation of nanograins to achieve non‐destructive locking between adjacent tablets for strain attenuation (Figure 12e),^[^ ^199^ ^]^ improved energy dissipation by nanograin rotation,^[^ ^85^ ^]^ torturous crack paths through intergranular organic matrix (Figure 12f),^[^ ^200^ ^]^ and enhanced frictional resistance against interfacial sliding from the nano‐asperities on nacre tablets (Figure 12g).^[^ ^30^, ^201^ ^]^

**Figure 13:**
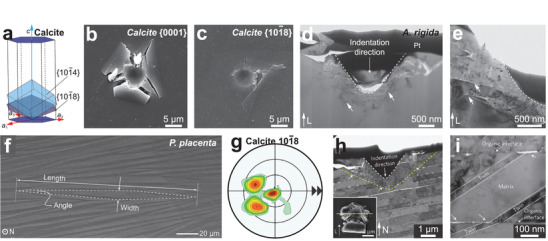
Mechanical dependence on structural orientation induced by crystallographic anisotropy. a) Schematic of hexagonal calcite unit cell, where the $\left\{10\bar{1}4\right\}$ and $\left\{10\bar{1}8\right\}$ rhombohedrons are illustrated. Fracture patterns on calcite b) {0001} and c) $\left\{10\bar{1}8\right\}$ planes by conical‐tip indentations. d,e) TEM images near the indentation zone in *A. rigida* prism, which show extensive cracks near the indent crater (white arrows). “L” denotes the length direction of the *A. rigida* prism. f) Top‐view SEM image of a freshly cleaved *P. placenta* shell, revealing the elongated diamond‐shaped laths. “N” denotes the shell normal direction. g) EBSD pole figure of *P. placenta*, showing almost coincidence of one $\left\{10\bar{1}8\right\}$ pole maxima with the shell surface. h,i) TEM images near the indentation zone in *P. placenta* shell, where i) the high‐resolution TEM image shows deformation twinning bands with parallel boundaries across the laths. The yellow dashed boundary in (h) encloses the plastic deformation region. “L” denotes the length direction of the laths. d,e) Reproduced with permission.^[^ ^28^ ^]^ Copyright 2020, Springer Nature. f) Reproduced with permission.^[^ ^45^ ^]^ Copyright 2013, John Wiley and Sons. g) Reproduced with permission.^[^ ^205^ ^]^ Copyright 2007, Elsevier. h,i) Reproduced with permission.^[^ ^206^ ^]^ Copyright 2014, Springer Nature.

Apart from the above discussion on the crystal texture difference, another important aspect is the intrinsic anisotropy of crystalline biominerals, where different crystallographic orientations exhibit different mechanical properties. For example, the indentation hardness measured from the measured $\left\{10\bar{1}4\right\}$, $\left\{10\bar{1}8\right\}$, and {0001} planes of calcite exhibit noticeable difference (Figure 2e). The stiffness anisotropy of calcite can be characterized by the complete stiffness matrix.^[^ ^202^, ^203^, ^204^ ^]^ The fracture behavior in calcite is also different when loaded on different planes. This is evidenced by the post‐indentation fracture patterns, where the {0001} surface shows the threefold cracking patterns (**Figure** **13**b) while the $\left\{10\bar{1}8\right\}$ surface exhibits anisotropic fracture with symmetric a cracking pattern (Figure 13c). Similarly, the inelastic deformation of biogenic calcite also exhibited drastic difference in these two crystallographic orientations. The *A. rigida* prism has the longitudinal axis parallel to the *c*‐axis of calcite, and nanoindentation loading along this direction induces brittle fracture with extensive cracks near the indentation craters (white arrows in Figure 13d,e).^[^ ^28^ ^]^ In comparison, the *Placuna placenta* shell has thin calcite lath‐like building blocks (Figure 13f)^[^ ^45^ ^]^ with the top surfaces parallel to the $\left\{10\bar{1}8\right\}$ planes (Figure 13g).^[^ ^205^ ^]^ Nanoindentation loading along the shell normal induced $\left\{01\bar{1}8\right\}$ twinning, which acted as effective boundaries to localize damage (Figure 13h,i).^[^ ^206^ ^]^ These examples demonstrate that the biogenic minerals with different crystallographic orientations contribute to specified elastic and fracture properties. In addition, the material level anisotropy may couple with the structural level anisotropy. For example, in the unique dual‐scale single‐crystalline microlattice found in starfish ossicles (*Protoreaster nodosus*), the stiffest [111] direction of its diamond microlattice structure compensates for the compliant *c*‐axis direction of calcite.^[^ ^207^ ^]^ The mechanical anisotropy of individual biomineral building blocks discussed here provides an additional strategy to control orientation‐dependent mechanical properties in the biological composites, which has been well recognized at the composite level.^[^ ^208^ ^]^

## 6 Residual Strain/Stress

Residual strains and stresses are involved in biominerals via a variety of origins, such as lattice distortions induced by intra‐OMs (Section 3), trace element substitutions (Section 4), and crystallization of ACC (Section 5). Residual stresses are defined as the stresses experienced by the material without applying external stress.^[^ ^36^ ^]^ In engineering materials, residual stresses could result from thermal (e.g., inhomogeneous cooling), chemical (e.g., nitriding in steels, new phase formation with volume shrinkage or expansion), or mechanical processes (e.g., non‐uniform plastic deformation). The effects of residual stress can be negative or positive. For the negative cases, for example, welding residual stress often induces premature brittle failure, especially under fatigue loading. On the other hand, for the beneficial cases, the compressive residual stress could strengthen the material's resistance to tensile loads. For example, laser peening introduces compressive residual stress to the material surface, which strengthens and toughens the material by delaying the crack opening (since cracks open by tensile stresses). Another good example is the tempered glass, which exhibits enhanced hardness and scratch resistance due to the compressive residual stress resulted from chemical doping.^[^ ^209^, ^210^, ^211^ ^]^

Based on their structural origins, three types of residual stress/strain have been reported in biomineral systems: i) Composite‐level residual stress/strain induced by structural mismatch between biominerals and inter‐OMs; ii) atomic‐scale residual stress/strain resulted from lattice distortions due to intracrystalline defects (intra‐OMs, Mg‐substitution, ACC, etc.); and iii) residual stress/strain caused by nano‐aggregation of trace elements in biomineral building blocks. The type‐i composite‐level residual stress can be released by mechanical destruction, whereas type‐ii and type‐iii biomineral‐level residual stresses at the atomic scale can be retained even after the biominerals are ground into powder form and will only be released when the atomic lattice is damaged through processes such as heat treatment. However, it is extremely challenging to directly characterize the mechanical influence from the residual stress in biominerals, especially for type‐ii and type‐iii. For instance, in preparation of control samples with residual stress removed, the associated processes such as, heat treatment leads to the change in compositions (e.g., intra‐OMs), which makes the comparison problematic.

### 6.1 Type‐i Residual Strain—Structural Mismatch at Composite Level

**Figure 14:**
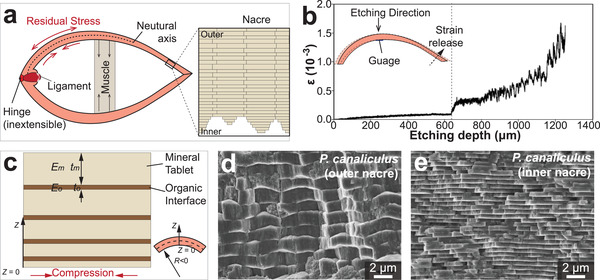
Residual strain/stress in biomineral composites at the composite level. a) Schematic of residual stress in bivalve shells, where the shell develops compressive residual stress in its inner layers and tensile stress in the outer layers.^[^ ^212^ ^]^ b) Strain release versus etched depth in *P. canaliculus* nacre, where the strain gauge was attached to the inner surface and etching was started from the outer surface. c) Schematic of the multilayered design in nacre structure with in‐depth change of tablet thickness, contributing to the strain gradient across the thickness direction. SEM images on the *P. canaliculus* nacre in the d) outer and e) inner region, respectively, confirming the in‐depth change of tablet thickness. b,d,e) Reproduced with permission.^[^ ^213^ ^]^ Copyright 2009, John Wiley and Sons.

The type‐i residual strain in biomineral composites was first observed by the change of curvature in bivalve shells after cutting, which resembled the behavior of strain release.^[^ ^212^ ^]^ Bivalve shells are subjected to stress throughout the animals’ life; the inner ligament is always under compressive stress while the adductor muscle and the outer hinge are under tensile stress, leading to elastic bending in the shells, that is, tensile stress in the shells’ outer region versus compressive stress in the inner (**Figure** **14**a). It was hypothesized that these strains were imprinted in the minerals during shell growth.^[^ ^212^ ^]^ Recent studies measured the strain release in the nacre layers of *P. canaliculus*, where the strain release increased gradually as a function of the etching depth and reached nearly 1.7 × 10^−3^ at the inner surface (Figure 14b).^[^ ^213^, ^214^ ^]^ This strain was too high to be produced by the muscular force, and an alternative origin of the residual strain was proposed, that is, the structural mismatch at the organic/inorganic interfaces (Figure 14c). Specifically, the strains in the mineral and organic layers, *ε*_m_ and *ε*_o_, are given as

(4) $$\varepsilon_{m}=\frac{\xi }{1+x},\varepsilon_{o}=-\frac{\xi x}{1+x},\mathrm{where}\;\;x=\frac{E_{m}t_{m}}{E_{o}t_{o}}$$

where the mineral and organic layers are indexed as “m” and “o,” respectively; *E*_m_ and *E*_o_ are their effective Young's moduli; *t*_m_ and *t*_o_ are the corresponding thicknesses; and *ξ* = *ε*_m_ − *ε*_o_ denotes the strain mismatch.^[^ ^213^ ^]^ For nacre layers, *E*_m_ >> *E*_o_, *t*_m_ >> *t*_o_, and thus *x* >>1. For the strained multilayered structure under elastic bending, the shell curvature *R* could be reproduced by strain gradients across the multilayers (*dε*/*dZ*),

(5) $$\varepsilon =-\frac{Z-t_{b}}{R}\Rightarrow \frac{d\varepsilon }{dZ}=-\frac{1}{R}$$

where *ε* corresponds to the strain at different depth; *t*_b_ is the position of neutral axis; and *Z* is the coordinate axis, where Z = 0 corresponds to the inner surface; and *R* is the radius of the concave shell (*R* < 0 due to coordinate definition, Figure 14c).^[^ ^213^ ^]^ By differentiating the strain in the mineral layers *ε*_m_ in Equation (4) using Equation (5), we have

(6) $$\frac{dt_{m}}{dZ}=\frac{xt_{m}}{\xi R}$$

Based on Equation (6), the strain gradients (*dε/dZ*) and the corresponding shell curvature (*1/R*) can be produced by either changing the compositional gradients (*E*_m_, *E*_o_, and therefore *x* and mismatch parameter *ξ*), or by gradual in‐depth changing of the tablet thickness *dt*_m_/*dZ*. Considering that the nacre layers were under compressive residual stress (*ξ* < 0), the corresponding tablet thickness gradient should be *dt*_m_/*dZ* > 0, that is, there should be a gradual increase of aragonite lamellae along the *Z*‐axis. This was confirmed by the SEM observations of the tablets near the outer and inner surface of the shell, respectively (Figure 14d,e).^[^ ^213^ ^]^

In addition to CaCO_3_‐based biominerals, residual stresses associated with the degree of hydration have also been reported in HAP/collagen‐based biomineral composites.^[^ ^215^ ^]^ For example, it was reported that dehydration could lead to contraction of collagen molecules and a corresponding compressive residual stress ≈300 MPa in the mineral phase of human dentin.^[^ ^216^ ^]^ It should be noted that the type‐i residual stress is originated at the composite level, which is not caused by intracrystalline features. However, the type‐i residual stress affects the strain level within biomineral units, and it is included here for a complete discussion of residual stresses in biominerals.

### 6.2 Type‐ii Residual Strain—Lattice Distortion at Atomic Scale

**Figure 15:**
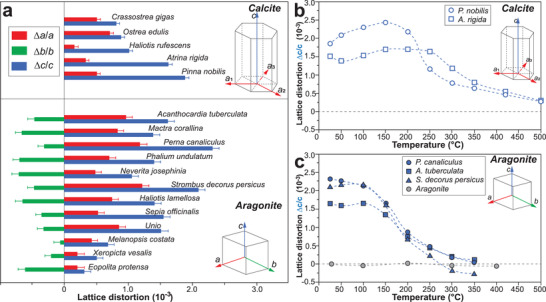
Lattice distortions in biogenic calcite and aragonite at the lattice scale. a) Summary of anisotropic lattice distortions in biogenic calcite and aragonite.^[^ ^68^, ^72^, ^73^ ^]^ The lattice distortion in calcite is described with the hexagonal system parameters ∆*a*/*a* and ∆*c*/*c*, while the aragonite is described with orthorhombic system parameters ∆*a*/*a*, ∆*b*/*b* and ∆*c*/*c*. Positive values indicate expansion (tensile strain), while negative values indicate shrinkage (compressive strain). b) Changes of Mg‐calcite *c*‐axis lattice parameters in *P. nobilis* and *A. rigida* as a function of annealing temperature, where lattice distortions were calculated with respect to geological calcite (after correction of Mg influence).^[^ ^217^ ^]^ c) Changes of aragonite *c*‐axis lattice parameters in *P. canaliculus*, *Acanthocardia tuberculata*, and *Strombus decorus persicus* as a function of annealing temperature, where lattice distortions were calculated with respect to geological aragonite.^[^ ^72^, ^73^ ^]^

The type‐ii residual strain originates from the lattice distortions of biomineral building blocks, which have been frequently reported in both biogenic calcite^[^ ^68^, ^217^ ^]^ and aragonite^[^ ^70^, ^71^, ^72^ ^]^ based on powder XRD measurements. **Figure** **15**a summarizes the measured lattice distortions in biominerals compared to geological calcite and aragonite.^[^ ^68^, ^72^, ^73^ ^]^ Strong orientation‐dependent lattice distortions have been observed, where the crystal lattices expand (positive strains) along the *a*‐axis and *c*‐axis in both calcite and aragonite systems, while the *b*‐axis of aragonite systems exhibits negative strains. Based on the elastic constants *C*_ij_ for calcite (rhombohedral system) and aragonite (orthorhombic symmetry), the corresponding residual stress can be calculated using the stiffness matrices

(7) $$\begin{array}{ccc} & & \left\{\begin{array}{c} \sigma_{11}=\sigma_{22}=\left(C_{11}+C_{12}\right)\frac{\Delta a}{a}+C_{13}\frac{\Delta c}{c}\\ \sigma_{33}=2C_{13}\frac{\Delta a}{a}+C_{33}\frac{\Delta c}{c} \end{array}\right.\;\mathrm{and}\;\;\\ & & \;\left\{\begin{array}{c} \sigma_{11}=C_{11}\frac{\Delta a}{a}+C_{12}\frac{\Delta b}{b}+C_{13}\frac{\Delta c}{c}\\ \sigma_{22}=C_{12}\frac{\Delta a}{a}+C_{22}\frac{\Delta b}{b}+C_{23}\frac{\Delta c}{c}\\ \sigma_{33}=C_{13}\frac{\Delta a}{a}+C_{23}\frac{\Delta b}{b}+C_{33}\frac{\Delta c}{c} \end{array}\right. \end{array}$$

for calcite and aragonite, respectively.^[^ ^186^ ^]^ The calculated maximum tensile residual stresses can reach 220 MPa in biogenic calcite (*P. nobilis*) and 182 MPa in biogenic aragonite (*P. canaliculus*) along their *c*‐axes.^[^ ^186^ ^]^ Such tensile stress may render the biominerals’ additional resistance to external compressive loadings (i.e., the loading must overcome the tensile residual stress first). It must be emphasized that the type‐ii residual strain in biominerals is defined based on the comparison with their geological counterparts using powder XRD, where averaged lattice parameters were obtained. However, the difference in the lattice parameters do not directly indicate absoulte value of the residual stress inside the biominerals, but instead the biogenic and geological minerals have different constituent lattices.

When heated above 200 °C, a significant lattice relaxation (strain release) was observed in both biogenic calcite and aragonite (Figure 15b,c).^[^ ^72^, ^73^, ^217^ ^]^ It was suggested that intra‐OMs may play a central role in the observed lattice distortions; the macromolecules decompose after heating, leading to lattice relaxation and corresponding strain release. However, slight differences between the biogenic calcite and aragonite should be noted: Mg substitution in calcite induces lattice shrinkage (see Section 4), which has been corrected for lattice distortion calculations in previous works (Figure 15b);^[^ ^68^ ^]^ while Mg substitution was insignificant in aragonite.^[^ ^104^ ^]^ In addition, a slight increase in calcite lattice parameters was observed as the biogenic calcite samples obtained from mollusk shells were slowly heated from room temperature to 200 °C (Figure 15b),^[^ ^217^ ^]^ which was not observed in aragonite systems (Figure 15c).^[^ ^72^, ^73^ ^]^ The underlying structural origin for this lattice expansion is currently unclear.^[^ ^68^ ^]^ A recent study on the echinoderm calcite found similar lattice expansions upon heating from room temperature to ≈200 °C (Figure 10j); without significant weight loss at this temperature range, the lattice expansion was attributed to the crystallization of the ACC phase.^[^ ^185^ ^]^

### 6.3 Type‐iii Residual Strain—Nano‐Aggregation of Trace Elements in the Coherent Mineral Matrix

The type‐iii residual strain is attributed to the nanoscale aggregation of trace elements within a biomineral matrix. Reported examples include Mg‐rich nanodomains in the Mg‐depleted calcite matrix of brittle star *O. wendtii* ^[^ ^24^, ^100^ ^]^ and Mg doping in human tooth enamel crystallites.^[^ ^218^ ^]^

**Figure 16:**
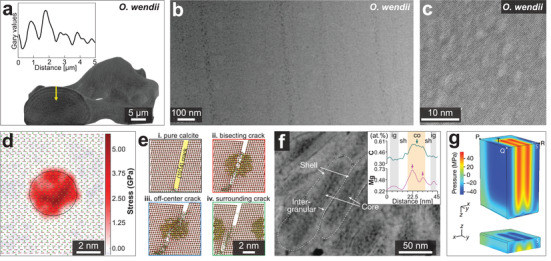
Residual stress due to nano‐aggregation of trace elements in the coherent mineral matrix. a–c) Multiscale characterization of Mg‐rich nanodomains in calcitic skeletal parts of brittle star (*O. wendtii*), including a) nano‐holotomographic (nano‐HT) reconstruction showing mass‐density oscillation along the thickness direction, b) TEM image showing the layered distribution of nanodomains (dark), and c) high‐resolution TEM revealing nanodomains (bright) coherently distributed in the biomineral matrix. d,e) MD simulations of a calcite crystal with an incorporated 40 mol% Mg nanodomain (diameter of 4 nm). d) The hydrostatic results indicate tensile stress (red) in the nanodomain and an angular compression in the matrix (light blue). e) Crack propagation under different conditions: i) Along the $\left\{10\bar{1}4\right\}$ cleavage plane in pure calcite, ii) bisecting the nanodomain, iii) slightly off‐center of the nanodomain, and iv) surrounding the nanodomain. f) STEM‐ADF image of enamel crystallites, where the arrows point to shell (sh) and core (co) regions of the crystallites, and the intergranular space (ig) between crystallites, respectively. The inset shows the trace element concentration profiles of C and Mg across an individual enamel crystallite. g) Finite element modeling of an enamel crystallite in equilibrium, showing that the core experiences residual tensile stress while the shell experience compressive stress. a–c) Reproduced with permission.^[^ ^100^ ^]^ Copyright 2019, Springer Nature. d,e) Reproduced with permission.^[^ ^219^ ^]^ Copyright 2020, Royal Society of Chemistry. f,g) Reproduced with permission.^[^ ^218^ ^]^ Copyright 2020, Springer Nature.

In the calcitic skeletal elements of brittle star *O. wendtii* (**Figure** **16**a–c), Mg‐rich nanodomains (≈40 mol% MgCO_3_) with the size of ≈5 nm are incorporated coherently in the Mg‐calcite matrix (≈13 mol% MgCO_3_).^[^ ^100^ ^]^ Nano‐holotomographic (nano‐HT) reconstruction (Figure 16a) and high‐resolution TEM imaging (Figure 16b) revealed that the Mg‐rich nanodomains have a layered distribution parallel to the skeleton surface with a periodicity of 0.7–1.5 µm.^[^ ^100^ ^]^ Such layered distribution of Mg‐rich nanodomains was achieved via the spinodal decomposition before crystallization, that is, a single phase Mg‐ACC transformed to a Mg‐rich phase and a Mg‐depleted phase.^[^ ^100^ ^]^ Since Mg‐rich nanodomains should have smaller lattice parameters than the matrix, it was inferred that the nanodomains experienced a hydrostatic elastic strain of ≈2.3% (tension) in contrast to the ≈−0.1% strain in the matrix.^[^ ^24^ ^]^ MD simulation of a nanodomain (4 nm in diameter) in calcite matrix showed a similar distribution of residual stress (Figure 16d).^[^ ^219^ ^]^ Further MD results revealed that the presence of these Mg‐rich nanodomains significantly modifies the fracture behavior of calcite (Figure 16e).^[^ ^219^ ^]^ In contrast to the $\left\{10\bar{1}4\right\}$ cleavage fracture in pure calcite (Figure 16e‐i), the crack bisected the nanodomain along a disordered path with significant bridging (Figure 16e‐ii). If off‐centered, the crack was either deflected when passing through the nanodomain (Figure 16e‐iii) or absorbed by the nanodomain (Figure 16e‐iv). The extracted stress‐strain curves from MD simulations also indicated that models with Mg‐rich nanodomains have a noticeable increase in strength.^[^ ^219^ ^]^ The layered distribution of the nanodomains can add another structural hierarchy, and contribute to toughening by crack deflection.^[^ ^24^ ^]^

In human tooth enamel crystallites, the selective distribution of trace elements has also been found to induce residual stress in HAP‐based biominerals.^[^ ^218^ ^]^ As shown in Figure 16f, the enamel crystallites have a sandwich‐like nano structure, with a core region rich in C (carbonate substitution) surrounded by Mg‐rich flanking. Based on finite‐element modeling, the enamel crystallite shows a similar residual stress distribution to the Mg‐calcite in brittle star, that is, a net tensile stress in the core and a compressive stress in the shell (Figure 16g).^[^ ^218^ ^]^ Such residual stress distribution, especially the compressive stress near the shell surfaces may impede crack initiation and thus increase toughness.

## 7 Twinning

Twinning can be considered as an intracrystalline feature with no addition of foreign components. Crystal twinning is defined as the different crystal domains joined together according to a specific symmetry operation, including reflection, rotation, and inversion.^[^ ^220^ ^]^ The corresponding mirror plane, rotation axis, and inversion center are defined as twin plane, twin axis, and twin center, respectively.^[^ ^221^ ^]^ There are three origins of twinning, that is, growth, transformation, and deformation (or glide):^[^ ^221^ ^]^ i) Growth twinning occurs when a new crystal is added to the face of an existing crystal, and the two crystals are oriented symmetrically by the shared face. ii) Transformation twinning occurs when a crystal undergoes a phase transformation due to a change in pressure or temperature; under the new pressure/temperature, a new crystal structure and symmetry is stable, which therefore promotes rearrangement of the original crystal to the new symmetry configuration. iii) Deformation twinning is produced when parts of a crystal are pushed out of place and form a symmetrical arrangement across a mirror plane. The shared plane by the parent crystal (matrix) and the newly formed crystal (twin) is defined as the twinning boundary (TB).

In metals, twinning can be introduced during growth (such as, electrodeposition and physical vapor deposition) or via deformation. Typically, increasing the density of twins (or decreasing spacing between twin planes) leads to the increase in strain hardening and strain rate sensitivity.^[^ ^222^, ^223^ ^]^ Moreover, recent research shows that nanotwins can act as strong barriers for dislocation motion, and therefore enhance the tensile strength and hardness of metals.^[^ ^222^, ^223^ ^]^ Compared with the classical strengthening strategies by introducing controlled internal defects (solute atoms, precipitates, and dispersed particles) and GBs, TB strengthening is often not accompanied with a sharp decrease in ductility.^[^ ^224^, ^225^ ^]^

**Figure 17:**
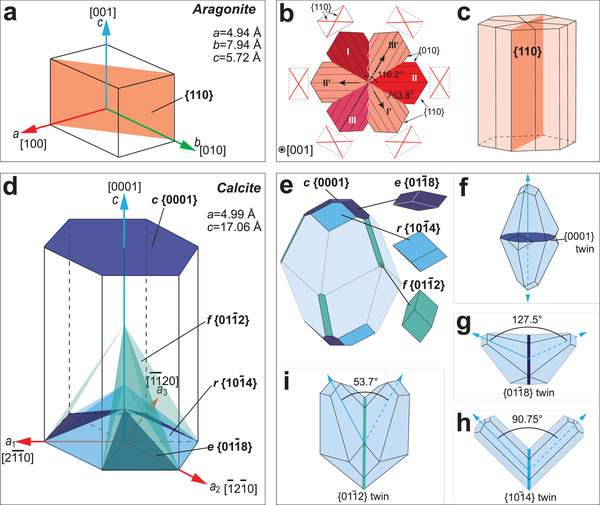
Twinning law in a–c) aragonite and d–i) calcite. a) Twinning plane (110) in relation to the aragonite crystal lattice. b) {110} growth twinning in aragonite induces the pseudohexagonal geometry of the aragonite crystal assembly when viewed down from [001] axis, where twin crystals II and III attach to the assembly by contact twin on (110) planes (red diagonal lines in the dashed aragonite crystals) to the master crystal I, and then the three crystals overgrow to the opposite to complete the inter‐penetration twinned crystals.^[^ ^226^ ^]^ c) 3D view of one (110) twinning plane in the pseudohexagonal aragonite crystal. d) Twinning planes in calcite, including *e* $\left\{01\bar{1}8\right\}$, *r* $\left\{10\bar{1}4\right\}$, *f* $\left\{01\bar{1}2\right\}$, and *c*{0001}, where each set of the twinning planes are marked with different colors. e) Schematic of a hypothetical calcite showing the four forms of twinning in calcite.^[^ ^227^ ^]^ f–i) Schematics of the typical morphologies of twinned calcite,^[^ ^227^ ^]^ where the *c*‐axes of the parent and twin are marked with solid and dashed blue arrows, respectively. The angles between the two *c*‐axes are the diagnostics of the twin laws.

**Figure** **17** summarizes the twin laws observed in geological aragonite and calcite, where aragonite twinning occurs across {110} twin planes (Figure 17a–c)^[^ ^226^ ^]^ and calcite twinning occurs on the *c*{0001}, *e* $\left\{01\bar{1}8\right\}$, *r* $\left\{10\bar{1}4\right\}$, and *f* $\left\{01\bar{1}2\right\}$ twin planes (Figure 17d‐i).^[^ ^227^, ^228^, ^229^ ^]^ Due to twinning, geological aragonite crystal often grows into a pseudohexagonal geometry when viewed along the [001] axis (Figure 17b,c):^[^ ^226^, ^230^, ^231^ ^]^ First, the contact twin II is developed by the reflection of the master crystal I on the (110) (dashed black line), with their *a*‐axes [100] meeting at an angle of 116.2°. The next twin crystal III then attaches to the assembly in the same manner. Later, the three crystals overgrow to the opposite, which produces the inter‐penetration twinned crystals with a 63.8° angle between each regular twinned pair. For twinned geological calcite, the shapes of the crystals can vary significantly, where the twin laws could only be recognized by the intersecting angles between two *c* axes (Figure 17f‐i).^[^ ^227^ ^]^ While growth twinning is more widespread in biogenic aragonite, deformation twinning is often observed in biogenic calcite.

### 7.1 Growth Twinning in Biogenic Aragonites

**Figure 18:**
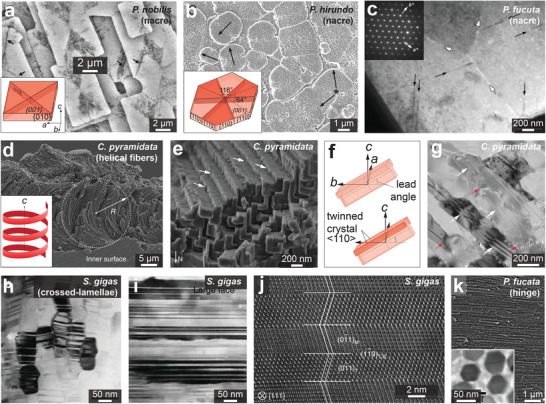
Growth twinning in aragonite‐based biominerals. a) SEM image of rectangular nacre platelets (*P. nobilis*) divided into four growth triangle sectors, where the intersecting boundaries of adjacent units are always parallel to the sides of the triangles (black arrows). b) SEM image of nacre platelets (*P. hirundo*), where the threefold orientations of the vermiculations (black arrows) indicate {110} twinning. c) Bright‐field TEM image of nacre tablets in (*P. fucata*), with the top‐left inset shows the electron diffraction pattern from the crystal domain at the bottom‐left. The tablet boundaries are marked by white arrows, and the contrasts of the thin twin individuals are marked by black arrows. d,e) SEM images of fractured *C. pyramidata* shells, showing the helical aragonite fibrous building blocks, where the white arrows in (e) show the variation in cross‐sectional geometries along the axial direction of fibers. f) Schematics show that the helical fiber growth is achieved by local alternation of preferred growth direction due to {110} twinning.^[^ ^238^ ^]^ g) TEM image of *C. pyramidata* aragonite fibers with twinning bands (white arrows). h,i) TEM images showing the high density of nanotwins in the third‐order lamellae of *Strombus gigas* shell, viewed from h) the end face and i) side surface of the prolonged lamellae, respectively. j) High‐resolution TEM image of nanoscale twins in the third‐order lamellae in *S. gigas* shell, where the TBs are coherent and parallel to the (1$\bar{1}$0) plane. k) SEM image of the hinge ligaments in bivalve *P. fucata*, showing the long aragonite fibers. The inset shows the TEM image of the twinned aragonite crystals in the hinge ligaments. a,b) Reproduced with permission.^[^ ^139^ ^]^ Copyright 2013, Elsevier. c) Reproduced with permission.^[^ ^232^ ^]^ Copyright 2012, Elsevier. d,e,g) Reproduced with permission.^[^ ^48^ ^]^ Copyright 2015, Springer Nature. h–j) Reproduced with permission.^[^ ^243^ ^]^ Copyright 2016, Springer Nature. k) Reproduced with permission.^[^ ^245^ ^]^ Copyright 2019, Elsevier.

**Figure** **18** summarizes some typical examples of twinned biogenic aragonites. The first thing to notice is that aragonite building blocks can be shaped into different geometries by twinning, and that twinning densities also vary significantly in different systems.^[^ ^232^, ^233^ ^]^ For example, the nacre tablets can be shaped into rectangular (*P. nobilis*, Figure 18a),^[^ ^139^ ^]^ pseudohexagonal (*Haliotis discus hannai*),^[^ ^234^ ^]^ or elongated irregular ellipse shapes (*P. hirundo*, Figure 18b).^[^ ^139^ ^]^ From the etched surface of nacre growth front, the vermiculations (parallel lineations of granular features) have three‐rayed distribution (black arrows in Figure 18b), which were confirmed to align parallel to the crystallographic *a*‐axis and therefore can be considered as an indication of twinning. TEM images on nacre tablets from the mollusk shell *P. fucata* showed very thin {110} twin domains, indicating a low density of twinning (Figure 18c).^[^ ^232^ ^]^

Growth twinning could be attributed to the organization patterns in the biomineral composites. Checa et al. found that the bivalve nacre crystals progressively oriented the tablets with their *b*‐axes parallel to the lateral growth direction, and this process was companied by the reduction of twinned tablets.^[^ ^235^ ^]^ Moreover, the unique epitaxial growth of foliated aragonite in *Micropilina arntzi* was also found to correlate with twinning.^[^ ^236^ ^]^

In addition, the fibrous aragonite building blocks were found to have high twinning densities, such as the helical aragonite fibers in *C. pyramidata* (Figure 18d–g)^[^ ^48^ ^]^ and *Cuvierina columnella*,^[^ ^237^, ^238^ ^]^ and the straight fiber‐like third‐order lamellae in a variety of crossed‐lamellar shells (Figure 18h–j).^[^ ^239^, ^240^, ^241^, ^242^, ^243^ ^]^ In particular, twinning was reported to facilitate the growth of helical fibers by abrupt changes of crystallographic growth direction (Figure 18f).^[^ ^238^ ^]^ Interestingly, Suzuki et al. proposed that the twinning density in biogenic aragonite is quantitatively correlated with the peak widths of XRD patterns, which was confirmed by the correlative TEM analysis on a wide range of biogenic aragonite samples.^[^ ^232^, ^233^ ^]^ In addition to the skeletal elements of mollusks, aragonite twinning has also been detected in other biomineralized components of different organisms. An intriguing example is the needle‐shaped aragonite crystals from the hinge ligament in molluscan bivalves, including *Mya arenaria*, *Spisula solidissima*,^[^ ^244^ ^]^ *P. fucata* (Figure 18k),^[^ ^245^ ^]^ *Neotrigonia* sp.,^[^ ^246^ ^]^ etc., where the thin twin domains transect the hexagonal fiber cross‐sections. Consider the functionality of the bivalve hinges, the extensively twinned aragonite crystals and the protein‐rich inter‐OMs might work in synergy for the extreme elasticity.^[^ ^245^ ^]^ Additional twinning examples include the corallite (*Galaxea fascicularis*) and the otolith of rainbow trout (*Oncorhynchus mykiss*).^[^ ^232^ ^]^

**Figure 19:**
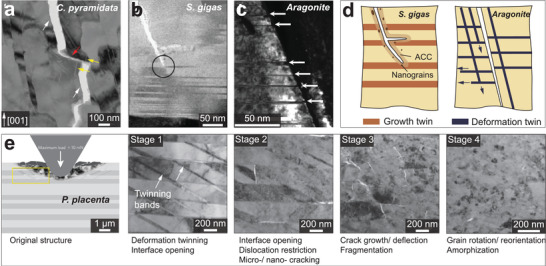
Mechanical contribution of twinning in biominerals. a) TEM image of the crack path across the building blocks in *C. pyramidata*, showing the intergranular (white arrows) and intragranular fracture (red arrow), as well as, the crack cutting through the {110} growth twins (yellow arrows). TEM images of b) biogenic aragonite (third‐order lamellae in *S. gigas* shell) showing the crack arrested by the growth twins (black circle) and c) geological aragonite showing nucleation of deformation twins from crack tip (white arrows). d) Schematic illustrations comparing the growth twinning in biogenic aragonite (left) and deformation twinning in geological aragonite (right). e) TEM images near the indentation crater illustrating the progression of nanoscale deformation mechanisms in *P. placenta* (biogenic calcite). a) Reproduced with permission.^[^ ^48^ ^]^ Copyright 2015, Springer Nature. b,c) Reproduced with permission.^[^ ^243^ ^]^ Copyright 2016, Springer Nature. e) Reproduced with permission.^[^ ^206^ ^]^ Copyright 2014, Springer Nature.

Recent works suggested that twinning contributes to the enhanced toughness of biominerals via crack deflection. TEM observation on *C. pyramidata* showed that TBs provided additional resistance by creating barriers along the fracture paths (**Figure** **19**a).^[^ ^48^ ^]^ The recent TEM study on the growth twinning in the 3rd‐order lamellae of *S. gigas* shells also confirmed the toughening mechanisms by crack deflection at TBs and phase transformation (nano‐grains and ACC) around the crack tip (Figure 19b).^[^ ^243^ ^]^ In comparison, extensive {110} twinning was also observed in geological aragonite, which does not hinder the cleavage cracks along (110) and ($1\bar{1}0$) planes (Figure 19c,d).^[^ ^243^ ^]^ In addition, deformation twinning was also observed in biogenic aragonite, which contributes to the increased high‐strain‐rate fracture strength of nacre.^[^ ^247^, ^248^ ^]^

### 7.2 Deformation Twinning in Biogenic Calcites

In biogenic calcite, deformation twinning could act as an important mechanism for damage resistance. In particular, our work on *P. placenta* (biogenic calcite laths) revealed that simultaneous efficient energy dissipation and damage localization can be achieved by the formation of $\left\{01\bar{1}8\right\}$ twinning near the damaged region.^[^ ^206^ ^]^ From the post‐indentation TEM images of *P. placenta*, the permanent deformation was confined in a V‐shaped zone enclosed by parallel twinning bands in each lath (Figure 19e). The deformation twinning boundaries then acted to restrict dislocation and promoted additional energy dissipation mechanisms, such as, nano‐cracking, fragmentation, and grain rotations, etc., while the damage is localized in the confined twinning boundaries (Figure 19e). Based on the calculated energy dissipation densities (energy dissipation divided by the volume of the damage zone), the *P. placenta* shell achieves a tenfold increase in energy dissipation density compared with single crystal calcite (0.290 ± 0.072 nJ µm^−3^ vs 0.034 ± 0.013 nJ µm^−3^).^[^ ^206^ ^]^

## 8 Conclusions and Outlook

### 8.1 Summary

Significant advances have been made recently to elucidate the internal microstructure and corresponding mechanical properties of biogenic minerals. In this review article, we focused on five important intracrystalline structural features, including intra‐OMs, trace elements, crystalline features, residual stress/strain, and twinning. These studies indicate that the biomineral building blocks conceal a rich library of design strategies for strengthening and toughening biominerals, which surpass the performance of their geological counterparts. As discussed in this review, some of these strengthening and toughening mechanisms have already been recognized in engineering structural materials, such as, precipitate strengthening (intra‐OMs), solid solution strengthening (trace elements and residual stress/strain), and twinning induced damage tolerance.

It should also be noted that some of the aforementioned structural features work synergistically to enhance the mechanical properties. For instance, intra‐OMs, ACC and Mg substitutions all contribute to the lattice distortions, that is, atomic‐scale residual strains.^[^ ^24^, ^36^, ^185^ ^]^ Moreover, some structural features often co‐exist, for example, ACC phases usually contain considerable trace elements including Mg^[^ ^150^ ^]^ and some intra‐OM molecules prefer binding with Ca to Mg^[^ ^249^ ^]^ and thus help stabilize the ACC phase.^[^ ^49^, ^96^ ^]^ Inclusion of Mg and organics is shown to reduce the strain required to induce twin formation in calcite.^[^ ^229^, ^250^ ^]^ An excellent example of biogenic mineral system with different intracrystalline mechanisms working in synergy is the grinding tip of sea urchin teeth: the Mg‐enriched polycrystalline calcite matrix improves wear‐resistance, while the Mg‐calcite single‐crystal calcite needles provide a relatively compliant framework, which together contribute to the self‐sharpening upon fracture.^[^ ^97^, ^98^, ^251^ ^]^

### 8.2 Open Questions

#### 8.2.1 Advanced Structural Characterizations

Despite significant progress in elucidating these intracrystalline structures in biomineral building blocks, there are still many open questions to be addressed, particularly in terms of atomic‐scale structural and chemical analysis, as well as 3D characterizations. For example, there has been no solid conclusion on whether the intracrystalline inclusions in biogenic minerals are completely filled with intra‐OMs, or whether any voids also present. The interaction between the intra‐OMs and the mineral matrix is crucial in understanding the mechanical properties of biogenic minerals; however, the detailed morphology of the organics‐mineral interfaces at atomic scale have not been fully revealed. Some unknown aspects include the 3D geometries of intra‐OMs, surface morphologies (rough or planar), chemical distribution within individual intra‐OMs, etc. Regarding the inorganic trace elements, atomic‐scale mapping of their distributions is currently not available. Recently, significant advancements have been made in atomic‐scale 3D imaging for synthetic nanomaterials under low beam damage conditions.^[^ ^252^, ^253^, ^254^ ^]^ In addition, high‐resolution 3D elemental mapping based on atomic probe tomography have been conducted for biogenic HAP crystals.^[^ ^218^, ^255^ ^]^ We believe that the application of advanced high‐resolution structural characterization will enable a deeper understanding of the intracrystalline ultrastructure and the corresponding mechanical properties of biominerals.

Furthermore, as most current studies focus on selected individual biomineral systems, we see a significant need and opportunity in conducting high‐throughput comparative structural/mechanical analysis for multiple biomineral systems. For example, so far, there have been a few case studies examining the detailed chemical compositions of intra‐OMs, such as that in calcite prisms^[^ ^44^, ^50^ ^]^ and porous echinoderm calcite.^[^ ^49^, ^51^ ^]^ Decoding the exact chemical composition of intra‐OMs in different biomineral systems might contribute to further finely tune the geometries, texture, and properties of bio‐inspired minerals.

#### 8.2.2 Formation Mechanisms

While the study of fully formed biominerals provides important insights for the structure‐property relationship, investigation of the structural evolution during the formation processes will offer important lessons for biomimetic synthesis and fabrication. There are many unanswered questions regarding how the intracrystalline nano‐/microstructures are formed. One example is related to the long‐range distribution of intra‐OMs, such as the gradual tilting of the intra‐OMs toward the inter‐OM boundaries in *A. rigida* prisms (Figure 4g);^[^ ^28^ ^]^ how this alignment is related to the mineralization front during growth is intriguing. In situ structural and chemical characterizations during growth are promising approaches to address this question. How trace elements are incorporated into the biogenic minerals can also be revealed by in situ chemical characterization. For example, whether they are from the mixture in the ACC precursor (one‐time incorporation) or continuous doping as the mineralization proceeds. Additionally, further understanding of crystallographic texture evolution in biomineral systems will benefit from the in situ structural characterization during growth. The commonly known examples are the prismatic calcite minerals, which follow a self‐similar structural evolution with their cross‐sectional morphologies fully predicted by classical materials science theories for normal grain growth;^[^ ^256^, ^257^ ^]^ yet, less is known regarding the evolution of the crystallographic properties of the individual biomineral building blocks (e.g., crystal splitting vs single‐crystal prisms).^[^ ^135^, ^188^, ^192^ ^]^

Twinning in biominerals presents another set of open questions related to growth. For biogenic aragonite, for example, there have been many observations of twinning in various aragonitic building blocks (Figure 18),^[^ ^232^, ^233^ ^]^ yet how twinning structures are formed and how twinning formation is related to the complex geometries of individual building blocks have not been fully understood. For biogenic calcite, despite the fact that growth twinning is commonly observed for geological calcite (*c*{0001}, *e* $\left\{01\bar{1}8\right\}$, *r* $\left\{10\bar{1}4\right\}$, and *f* $\left\{01\bar{1}2\right\}$, Figure 17d‐i),^[^ ^227^ ^]^ there has been no report on the growth twinning in biogenic calcite. The underlying reason for such difference in growth twinning for biogenic calcite, geological calcite, and biogenic aragonite is intriguing. Finally, further investigation on the twinning structures from other biominerals, such as HAP crystals in teeth systems,^[^ ^258^ ^]^ may provide further insights into their formation pathways and mechanical properties.

#### 8.2.3 Optimization or Byproduct?

It is generally accepted that biogenic minerals exhibited improved mechanical properties than their geological counterparts, particularly at the composite level (see our companion review article).^[^ ^19^ ^]^ However, it is still an open question whether the intracrystalline structural features of biogenic minerals (such as the presence of intra‐OMs, aggregated nanodomains, growth twinning, etc.) are optimized during course of evolution or simply the “byproduct” during growth. For example, for the intra‐OMs incorporated into biominerals from the biologically controlled biomineralization process, although enhancement of the mechanical properties (such as the hardness and strength) has been observed, it is challenging to conclude such a structure is “optimized” for better performance. More insights in this direction may be gained from the investigation of the biomineralized skeletons from the historical records. Recently, Gilbert et al. revealed that the early biominerals from Ediacaran (≈550 Ma) and Cambrian (≈500 Ma) shelly fossils show similar nanoparticulate texture with modern biominerals, indicating that the same thermodynamics and kinetics may be responsible for biomineralization, that is, crystallization by particle attachment of amorphous precursors.^[^ ^259^ ^]^ In addition, systematic parametric computational modeling may also provide further understanding by studying the effects of, for instance, the geometries, distributions, alignments, and compositions of the intracrystalline microstructures in different biomineral systems.

#### 8.2.4 Amorphous Biominerals

Apart from the transient amorphous phases (e.g., ACC in Section 5.2), stable amorphous biominerals are also found in organisms, particularly the amorphous biogenic silica. Examples include the spicules with concentric layers in sea sponges,^[^ ^260^, ^261^ ^]^ the complex frameworks in diatoms and radiolaria,^[^ ^146^ ^]^ and the nanoparticles distributed in bamboo skin.^[^ ^262^ ^]^ Intracrystalline features in the biosilica building blocks have been reported, including intra‐OMs^[^ ^263^ ^]^ and trace elements (Na, C, etc.).^[^ ^264^ ^]^ Currently, detailed investigation on the mechanical effects of the intracrystalline microstructures in biogenic silica is limited. For example, the gradient decrease in indentation hardness and modulus was observed from the core to the periphery of sea sponge spicules,^[^ ^265^ ^]^ however, the underlying structural origin is currently not understood. In addition, how the degree of hydration within the silica matrix regulates the local mechanical properties in biogenic silica is unclear.^[^ ^266^ ^]^ The continuous progress in this direction may provide further insights for the synthesis of bio‐inspired silica‐based materials.

#### 8.2.5 Bio‐Inspired Synthesis

With increasing interests in the research of biological materials, one ultimate goal is to fabricate bio‐inspired high‐performance engineering materials. Most of the current progress on bio‐inspired material designs concentrates on mimicking the final structures of the biological materials,^[^ ^4^ ^]^ while there is less attention and emphasis on the bio‐inspired synthesis and fabrication. Many current structural mimicking designs require scaling‐up fabrications of the micro‐/nanostructures observed in biological materials, which often lead to reduced hierarchies. In addition,much attention is directed to engineer the composite structure instead of the internal microstructure of the reinforced particles, which are usually ceramic‐ or metal‐based and used to mimic the mineral building blocks in biomineralized composites. The weakened constituent materials limited by the available fabrication techniques lead to degraded properties of biomimetic composites. For instance, metals or ceramics based additive manufacturing techniques have been applied to replicate the hard phases of the biomineral composites, but the printed products are usually weaker than the corresponding solid constituents due to defects (e.g., porosities, inhomogeneity, sintering defects) induced during the printing or post‐treatment process. In addition, many manufacturing processes usually require high temperature and/or pressure conditions.

Bio‐inspired synthesis may provide alternative approaches to address the aforementioned issues. One promising example is the bio‐inspired synthesis of calcite with incorporation of organic materials (e.g., copolymer micelles, amino acids, and polystyrene particles),^[^ ^74^, ^75^, ^267^ ^]^ where the synthetic calcites showed comparable hardening and toughening behavior to biogenic calcite. There has also been extensive work on bio‐inspired synthesis of Mg‐containing calcite crystals by introducing additives to stabilize ACC precursors.^[^ ^96^ ^]^ Typical additives include polyacrylic acids,^[^ ^268^ ^]^ carboxylic acids,^[^ ^249^, ^269^ ^]^ polypeptides,^[^ ^111^ ^]^ polysaccharides,^[^ ^270^ ^]^ and organics extracted from biominerals.^[^ ^49^, ^271^ ^]^ Regarding the size and geometric control, polymers with different chain lengths and structures can be used as the intracrystalline inclusions.^[^ ^74^, ^75^, ^267^ ^]^ Further investigations on the organics‐mineral interface will provide additional insights to precisely control the incorporation of organic inclusions in synthetic material systems.^[^ ^272^ ^]^ For the orientation and distribution control of intra‐OMs, modulating the growth media dynamically by mimicking the day/night and seasonal cycles in the mollusk shell formations may offer promising routes.^[^ ^273^, ^274^, ^275^ ^]^ Last but not least, as the current approaches to fabricate nano‐twinned metals requires high temperature and pressure,^[^ ^222^, ^223^, ^276^ ^]^ it is highly beneficial to study and learn from the twinning formation mechanisms in biogenic aragonite.^[^ ^232^, ^233^ ^]^

## Conflict of Interest

The authors declare no conflict of interest.
